# Simultaneous serotonin and dopamine monitoring across timescales by rapid pulse voltammetry with partial least squares regression

**DOI:** 10.1007/s00216-021-03665-1

**Published:** 2021-10-23

**Authors:** Cameron S. Movassaghi, Katie A. Perrotta, Hongyan Yang, Rahul Iyer, Xinyi Cheng, Merel Dagher, Miguel Alcañiz Fillol, Anne M. Andrews

**Affiliations:** 1 Department of Chemistry & Biochemistry, University of California, Los Angeles, Los Angeles, CA 90095 USA; 2 California NanoSystems Institute, University of California, Los Angeles, Los Angeles, CA 90095 USA; 3 Department of Psychiatry and Biobehavioral Sciences, Semel Institute for Neuroscience and Human Behavior, and Hatos Center for Neuropharmacology, University of California, Los Angeles, Los Angeles, CA 90095 USA; 4 Department of Electrical Engineering, University of California, Los Angeles, Los Angeles, CA 90095 USA; 5 Molecular Toxicology Interdepartmental Program, University of California, Los Angeles, Los Angeles, CA 90095 USA; 6 Interuniversity Research Institute for Molecular Recognition and Technological Development, Universitat Politècnica de València - Universitat de València, Camino de Vera s/n, 46022 Valencia, Spain

**Keywords:** Neurotransmitters, Electrochemistry, Brain, In vivo, Machine learning

## Abstract

Many voltammetry methods have been developed to monitor brain extracellular dopamine levels. Fewer approaches have been successful in detecting serotonin in vivo. No voltammetric techniques are currently available to monitor both neurotransmitters simultaneously across timescales, even though they play integrated roles in modulating behavior. We provide proof-of-concept for rapid pulse voltammetry coupled with partial least squares regression (RPV-PLSR), an approach adapted from multi-electrode systems (i.e., electronic tongues) used to identify multiple components in complex environments. We exploited small differences in analyte redox profiles to select pulse steps for RPV waveforms. Using an intentionally designed pulse strategy combined with custom instrumentation and analysis software, we monitored basal and stimulated levels of dopamine and serotonin. In addition to faradaic currents, capacitive currents were important factors in analyte identification arguing against background subtraction. Compared to fast-scan cyclic voltammetry-principal components regression (FSCV-PCR), RPV-PLSR better differentiated and quantified basal and stimulated dopamine and serotonin associated with striatal recording electrode position, optical stimulation frequency, and serotonin reuptake inhibition. The RPV-PLSR approach can be generalized to other electrochemically active neurotransmitters and provides a feedback pipeline for future optimization of multi-analyte, fit-for-purpose waveforms and machine learning approaches to data analysis.

**Graphical abstract:**

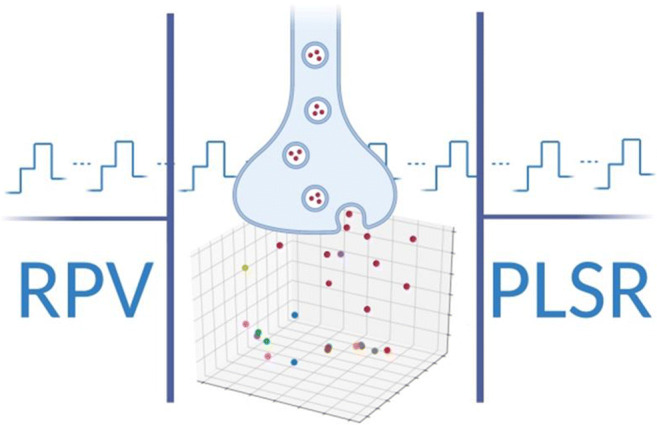

**Supplementary Information:**

The online version contains supplementary material available at 10.1007/s00216-021-03665-1.

## Introduction

The idea that neurotransmitters function via coordinated activities to shape behavior is becoming increasingly supported by in vivo studies [1–9]. We recently found that optogenetic stimulation of midbrain dopamine neurons, which drives reward-related behavior [10], produces serotonin release in striatum [11]. Dopamine and serotonin neurons directly and indirectly form circuits with one another [12–14]. Both systems exhibit developmental, functional, and clinical interplay [15, 16]. The dopamine and serotonin systems are implicated in diverse behaviors of relevance to neuropsychiatric and neurological disorders, including major depressive and anxiety disorders [17, 18], schizophrenia [19, 20], substance use disorder [21, 22], and Parkinson’s disease [23, 24]. These and other findings support the overarching hypothesis that multiple neurochemical systems, and particularly, the dopamine and serotonin systems, function (or dysfunction) concertedly [25–27].

Neurochemical signaling encodes biologically relevant information across multiple timescales [28]. Tonic (basal) neurotransmitter levels arise from clocklike neural firing over minutes to hours to days. Phasic (transient) changes in neurotransmitter levels are rapid (tens of milliseconds to seconds) and are hypothesized to result from synchronized bursts of neural firing in response to evoked or naturally occurring stimuli [29–33]. The ability to monitor transitory neurochemical events, in conjunction with changes in tonic signaling, will enable a more comprehensive understanding of how chemical neurotransmission encodes behaviorally relevant information [34, 35].

A variety of techniques are available for in vivo neurochemical monitoring with various advantages and disadvantages [36–39]. Here, we focus on voltammetry methods, including fast-scan cyclic voltammetry (FSCV), to detect electroactive neurotransmitters. The use of small carbon fiber microelectrodes (5–30 μm diameter) [40, 41] and high sampling rates (10–100 Hz) [42, 43] in FSCV can be used to differentiate release vs. reuptake processes [44]. While widely employed, FSCV suffers from poor analyte specificity. Overlapping oxidation (and reduction) profiles of structurally similar neurochemicals, many of which occur at low concentrations, make in vivo measurements of transmitters other than dopamine difficult with FSCV [45]. Moreover, FSCV is limited by the need for background subtraction of large capacitive currents generated during voltage sweeps at fast-scan rates. Background subtraction precludes tonic (basal) neurotransmitter determinations and measurements over longer time frames, (e.g., minutes-hours), due to current drift [46, 47].

Several novel waveforms have been developed that improve and expand various aspects of sweep-wave voltammetry [42, 48]. Fast-scan controlled absorption voltammetry (FSCAV) enables determination of basal dopamine or serotonin levels [34, 49, 50]. Other adsorption waveforms and accumulation electrodes have been reported [51, 52]. Meunier et al. [53] devised a waveform that allowed prediction and subtraction of electrochemical drift for measurements of dopamine, adenosine, and H_2_O_2_, as well as sweep waveforms to detect the opioid peptide met-enkephalin, H_2_O_2_, and pH [54, 55].

Complex waveforms that combine sweeps or staircases with square-wave pulses have been reported. Multiple cyclic square-wave voltammetry was used to quantify tonic dopamine in vivo with 10-s resolution [56]. Improvements in selectivity and sensitivity were made using fast-cyclic square-wave voltammetry (FCSWV) [57] and N-FCSWV [58] for monitoring dopamine and serotonin in vivo, respectively. Multiplexing has not yet been achieved with square-wavevoltammetry—two different waveforms were needed to measure dopamine [57] vs. serotonin [58]*.* Additionally, capacitive current simulation, which relies on assumptions about exponential current decay, was needed for background subtraction. Swamy and Venton [59] used single-walled carbon nanotube electrodes with FSCV to measure simultaneous changes in dopamine and serotonin in vivo. The carbon nanotube coating reduced the formation of oxidative byproducts of serotonin and increased the cathodic currents of dopamine and serotonin, improving analyte discrimination via more distinct reduction profiles [60].

Principal components analysis (PCA) [61] and principal components regression (PCR) [62, 63] have been used for multiplexing via dimensionality reduction in FSCV, with PCR capable of quantitative predictions. Another dimensionality reduction method widely used in chemometrics is partial least squares regression (PLSR) [64]. The PLSR approach is a supervised machine learning technique (i.e., it models input and output); PCA and PCR are considered unsupervised (i.e., only input data is modeled). The use of PLSR was shown to improve predictive accuracy over PCR when analyzing FSCV data for mixtures of neurochemicals [65]. Other uses included prediction and correction of FSCV background drift and pH changes [53, 55]. Kishida et al. [66, 67], Bang et al. [68], and Moran et al. [69] pioneered combining FSCV with regularized linear regression (i.e., elastic net electrochemistry) for subsecond monitoring of evoked dopamine [66, 67] and serotonin [68, 69] in the human striatum during decision-making tasks.

While newer waveforms and data processing methods have advanced neurochemical measurements, no single voltammetry technique yet enables tonic and phasic levels of multiple neurotransmitters to be determined simultaneously. To address this, we demonstrate a two-pronged approach to improve waveform design and data analysis. We gained inspiration from the voltammetric electronic tongue (VET) [70], used to measure analytes in food [71, 72], beverages [73, 74], and wastewater [75]. Rather than using conventional pulse waveforms, “smart” pulse waveforms are designed for VET sensing. These pulse trains are initially constructed based on the electrochemical characteristics of the analytes of interest [76]. Pulse widths and amplitudes, as well as pulse train frequencies, among other factors, are optimized to extract distinguishing electrochemical characteristics for data processing [77, 78]. Smart pulse design has been shown to outperform conventional [76] and random [71] pulse waveforms using the VET method.

Data generated by the VET method have been analyzed using a multivariate technique, commonly PLSR [72, 74]. As PLSR models covariance, the model prioritizes variations in input (current response) that correspond to qualitative and quantitative changes in output (analyte classification and concentration) [64]. As such, differences in the Helmholtz double layer, mass transport, analyte concentrations and adsorption, and other dynamic electrode surface properties occurring during an applied pulse are considered potential sources of analyte-specific information. This information is encoded in the transient responses of faradaic and non-faradaic currents. By including faradaic and non-faradaic current responses as input to the model (i.e., not background subtracting), the PLSR model selects aspects of the current response that covary with analyte identity and concentration. This is opposed to background-subtracted methods, where some information is discarded prior to model input to increase signal-to-noise. Potentially relevant information in the background is then lost.

An appropriately trained model can handle voltammetry data without the need for background subtraction, noise filtering/removal, or drift subtraction. In addition to VET studies, regularized regression applied to FSCV has been used to demonstrate that appropriately trained models benefit from information beyond analyte redox potentials when background subtraction is avoided [67, 69]. The use of regularized regression accounted for drift and noise, similar to PLSR.

Here, we report on the initial development of rapid pulse voltammetry coupled with PLSR (RPV-PLSR) using a smart pulse approach. By avoiding background subtraction, RPV-PLSR utilizes faradaic and non-faradaic current to improve analyte identification and quantification power. Inclusion of the background current also enables tonic and phasic concentration predictions in a single experiment at fast timescales (i.e., limited only by waveform frequency).

## Materials and methods

### Chemicals

Dopamine hydrochloride (#H8502) and serotonin hydrochloride (#H9523) were purchased from Sigma-Aldrich (St. Louis, MO). Artificial cerebrospinal fluid (aCSF) for in vitro experiments consisted of 147 mM NaCl (#73575), 3.5 mM KCl (#05257), 1.0 mM NaH_2_PO_4_ (#17844), 2.5 mM NaHCO_3_ (#88208) purchased from Honeywell Fluka (Charlotte, NC), and 1.0 mM CaCl_2_ (#499609) and 1.2 mM MgCl_2_ (#449172) purchased from Sigma-Aldrich. The aCSF solution was adjusted to pH 7.3 ± 0.03 using HCl (Fluka, #84415). The phosphate-buffered mobile phase for high-performance liquid chromatography (HPLC) consisted of 96 mM NaH_2_PO_4_, 3.8 mM Na_2_HPO_4_ (Fluka #71633), pH 5.4, 2–2.8% MeOH (EMD #MX0475), 50 mg/L EDTA·Na_2_ (Sigma #03682), and 500 mg/L sodium decanesulfonate (TCI #I0348) in water. All aqueous solutions were made using ultrapure water (Milli-Q, Millipore, Billerica, MA).

### In vitro experiments

For in vitro training data used for preliminary method validation, carbon fiber microelectrodes were fabricated as described previously [41] with minor modifications. Single 7-μm-diameter carbon fibers (Specialty Materials, Lowell, MA) were vacuum-aspirated into borosilicate glass capillaries (Sutter Instrument Company, Novato, CA). Each capillary was pulled to produce two electrodes by tapering and sealing using a micropipette puller (P-1000, Sutter Instrument Company, Novato, CA). Electrode tips were cleaned with 100% isopropanol (Fisher A416P, for electronic use) for 10 min and dried at 90–100 °C for 10–20 min. Electrode tips were then sealed by dipping in non-conductive epoxy (Epoxy Technology Inc., Billerica, MA) for 7–10 min twice at a 1-h interval at room temperature. Epoxied electrodes were dried at 90–100 °C overnight. Prior to testing, electrode tips were blunt-cut using a surgical scalpel under a microscope to create 7-μm-diameterdisk-shaped conducting surfaces. Bare silver wire (0.010-in. diameter, A-M Systems, Sequim, WA) was cleaned using a polishing cloth and inserted into working electrode capillaries to serve as the electrical connection (Fig. S1). The electrodes were backfilled with 2 M aqueous NaCl for electrical connection. Reference electrodes (RE-5B Ag/AgCl, BASi, West Lafayette, IN) used for all in vitro experiments were maintained in oversaturated aqueous KCl. Fresh aCSF was delivered to a flow cell at a constant flow rate of 2.5–2.7 mL/min by a peristaltic pump (Fig. S1). Standards (180 μL) of dopamine, serotonin, and their mixtures were injected via an autoinjector (VICI E60 Actuator, Valco Instruments Co. Inc., Houston, TX) in pseudo-random order at >5-min intervals.

### In vivo experiments

#### Animals

Subjects were virgin female mice generated at the University of California, Los Angeles (UCLA) from a DAT^IRES*cre*^ lineage (Jackson Laboratory, stock no. 006660) on a C57Bl/6J background via heterozygote mating. All surgeries were carried out under aseptic conditions with isoflurane anesthesia (5% isoflurane for induction, 1.5–2% for maintenance) on a KOPF Model 1900 Stereotaxic Alignment System (KOPF, Tujunga, CA). After each surgery, mice were administered the analgesic carprofen (5 mg/kg, 1 mg/mL, sc) for the first 3 days, and an antibiotic (amoxicillin, 0.25 mg/mL) and analgesic (ibuprofen, 0.25 mg/mL) in drinking water for 14 days post-surgery.

#### RPV-PLSR

Three mice first underwent a surgical procedure for head-bar implantation. A pair of rectangular head-bars (9 mm × 7 mm × 0.76 mm, 0.6 g each, laser cut from stainless steel at Fab2Order) were attached to the sides of the skull by C&B-METABOND (Fig. S1; Parkell, Edgewood, NY). The Cre-dependent adeno-associated viral vector (AAV) was obtained from the University of North Carolina Vector Core (Chapel Hill, NC). A nanoinjector was used to deliver 600 nL of 7.8 × 10^12^/mL AAV5/Syn-Flex-ChrimsonR-tdT unilaterally into the ventral tegmental area (VTA)/substantia nigra (SN) area (AP −3.08 mm, ML ±1.20 mm, DV −4.00 mm from Bregma). Then, a 200 μm diameter ferrule-coupled optical fiber (0.22 NA, Thorlabs, Newton, NJ) was implanted (AP −3.08 mm, ML ±1.20 mm, DV −3.80 mm from Bregma via the same path of viral vector injection) to deliver optical stimulation during experiments.

After surgery, mice were pair-housed with cagemates to recover for at least 2–3 weeks and to allow for expression of genes of interest before an additional craniotomy surgery. During this time, subjects were trained to acclimate to the head-fixed testing condition for 15–30 min/session × 6–10 sessions. Two craniotomies were carried out 24 h ahead of testing days. A piece of the skull (2.0 mm width × 2.0 mm length, centered at AP +1.0 mm, ML ±1.0 mm from Bregma) above the striatum (STR) of the same hemisphere as the AAV injection site was removed for working electrode insertion. For the Ag/AgCl reference electrode, a 0.4-mm diameter hole (centered AP +2.8 mm, ML ±2.0 mm from Bregma) was made in the skull on the side contralateral to the AAV injection site. The dura remained intact for both surgical areas. All surgery areas were first sealed with a thin layer of Kwik-Cast & Kwik-Sil (World Precision Instruments, Sarasota, FL) and then covered with a thin layer of C&B-METABOND. Animals were allowed to recover for 24 h.

On the testing day, each mouse was transferred and mounted to the head-fixed stage via its head-bars (Fig. S1). After a 10-min habituation period, the C&B-METABOND cover, Kwik-Cast & Kwik-Sil seal, and dura above the recording and reference electrode sites were carefully removed. A Ag/AgCl reference electrode made from bleached silver wire was lowered into the brain. An optical fiber was calibrated to 10 mW/mm^2^ daily prior to fiber coupling. Optical stimulation was generated via a 532 nm MGL-III-532 laser (Changchun New Industries Optoelectronics Tech. Col, Ltd., Changchun, People’s Republic of China). Square pulses of 50% duty at 30 or 40 Hz for 20 s were used to deliver optical stimulation at >5-min intervals. One subject received a dose of escitalopram (20 mg/kg, sc). Basal and optically stimulated responses were collected before and beginning 1 h after drug administration.

The working electrode (PEDOT:Nafion carbon fiber microelectrode) was sterilized using 70% ethanol, rinsed with saline, and lowered into the striatum for voltammetry measurements via a 1-μm precision motorized digital micromanipulator (MP-225, Sutter Instrument, Novato, CA). The PEDOT:Nafion-coated electrodes were fabricated as per published protocols [79]. Each electrode had a cylindrical conducting surface that was 5 μm in diameter and ~ 75 μm in length. When lowered to a new recording depth, the electrode baseline was restabilized for at least 10 min before continuing stimulations.

During testing, sweetened condensed milk diluted with water was delivered to the subject every 2 h. Subject behavior was monitored for signs of distress. After the experiment, each subject was prepared for histological verification of Chrimson expression, recording electrode position, and the position of the optical fiber. At the end of each in vivo experiment, electrodes were removed and post-calibrated using standards of dopamine, serotonin, and their mixtures in physiological saline to generate the training set data.

#### Microdialysis

Mice (*N* = 3) at 3–6 months of age were Chrimson-transfected, had an optical fiber implanted, and were trained to be head-fixed, as described above. Two to three weeks after Chrimson transfection, a second surgery was carried out to implant a CMA/7 guide cannula for a microdialysis probe aimed at the dSTR (AP + 1.00 mm, ML ± 1.75 mm, DV −3.10 mm from Bregma) into the same hemisphere as the viral delivery and fiber implant site (see above). The guide cannula was secured to the skull with C&B-METABOND. Animals recovered from the surgery for at least 3 days before microdialysis. Subjects underwent online microdialysis testing for 1 day. Following testing, the microdialysis probe was removed and the brain of each mouse was prepared for histology to verify the microdialysis probe and optical fiber placements, and Chrimson expression. Microdialysis probe and optical fiber tracks were visualized using light microscopy.

On the night before microdialysis (ZT10–12), each mouse was briefly anesthetized with isoflurane (1–3 min) for insertion of a CMA/7 microdialysis probe (1 mm length, 6 kDa cutoff, CMA8010771; Harvard Apparatus, Holliston, MA) into the guide cannula. Subjects were returned to their home cages after insertion and aCSF was continuously perfused through the probe at 2–3 μL/min for 30–60 min followed by a 0.3 μL/min flow rate for an additional 12–14 h to allow stabilization of the brain tissue surrounding the probe.

On the testing day, subjects were relocated to the headstage recording setup and allowed to habituate for at least 30 min before basal data collection. Optical stimulation was performed as described above, except the pulses were delivered at 10 Hz for 5 min. The first stimulation was delivered at ~ZT-2 after 6–18 basal dialysate samples were collected and analyzed. Prior to reverse dialysis of escitalopram (10 μM), three optical stimulations were delivered at 1-h intervals. After 90–120 min of intrastriatal drug perfusion, an additional three optical stimulations were delivered at 1-h intervals while drug perfusion was continued.

High-performance liquid chromatography was performed using an Amuza HTEC-500 integrated system (Amuza Corporation [formally known as Eicom], San Diego, CA). An Eicom Insight autosampler was used to inject standards and Eicom EAS-20s online autoinjectors were used to collect and inject microdialysis dialysates [80]. Chromatographic separation was achieved using an Eicom PP-ODS II column (4.6 mm ID × 30 mm length, 2 μm particle diameter). The column temperature was maintained at 21 °C. The volumetric flow rate was 450–500 μL/min. Electrochemical detection was performed using an Eicom WE-3G graphite working electrode with an applied potential of +450 mV vs. a Ag/AgCl reference electrode.

Standard curves encompassed physiological concentration ranges of serotonin and dopamine in dialysates (0–10 nM). The limit of detection was ≤300 amol (6 pM) for each analyte; the practical limit of quantification was ≤900 amol (20 pM). Dialysate samples were collected online at 5-min intervals using a dialysate flow rate of 1.8 μL/min and injected immediately onto the HPLC system for analysis.

### Voltammetry data acquisition and analysis

#### Measurement hardware

Voltammetry measurements were carried out using a two-electrode configuration via a Ag/AgCl reference electrode and a carbon fiber microelectrode working electrode. Waveforms were generated using a PC with a PCI-6221 data acquisition card (National Instruments (NI), Austin, TX) to control an EI-400 potentiostat (Cypress Systems, USA) and a custom “headstage” analog pre-amplifier. Potentials were applied to the reference electrode while the working electrode was tied to the zero-potential terminal (virtual ground) of the pre-amplifier circuit. The pre-amplifier was designed to output an analog voltage proportional to electrode current. Detailed information on the custom headstage design is in the Supplemental Information (Fig. S2). The output voltage was amplified by the EI-400, then sampled and quantified by an analog-to-digital converter on the NI PCI-6221 data acquisition card.

#### Measurement software

An in-house software program was developed for this study. The software was programmed in MATLAB (R2016a; The MathWorks, Inc., Natick, MA) and consisted of three modules. (1) The signal generation module enabled the design of multi-step waveforms at user-specified potentials, scan rates, sampling and fundamental frequencies, and numbers of sampled points per waveform period. (2) The MATLAB Data Acquisition Toolbox enabled event-driven communication during the measurement process. Waveforms were loaded from the signal generation module while the user specified the measurement start and stop points, along with optional parameters for stimulation or injection events. The data acquisition card generated the analog potential signal and the stimulation signal and digitized the resulting current. Voltammograms for each measurement cycle and the temporal evolution of current at potentials of interest were plotted in real time. At the end of each measurement, digitized current measurement data were stored in MATLAB files. (3) The data processing module displayed the acquired data in a variety of user-specified formats, allowed for user-defined background subtraction, digital filtering and signal averaging, and generated MATLAB or Excel files to be extracted for machine learning models.

#### Waveforms

**Fig. 1:**
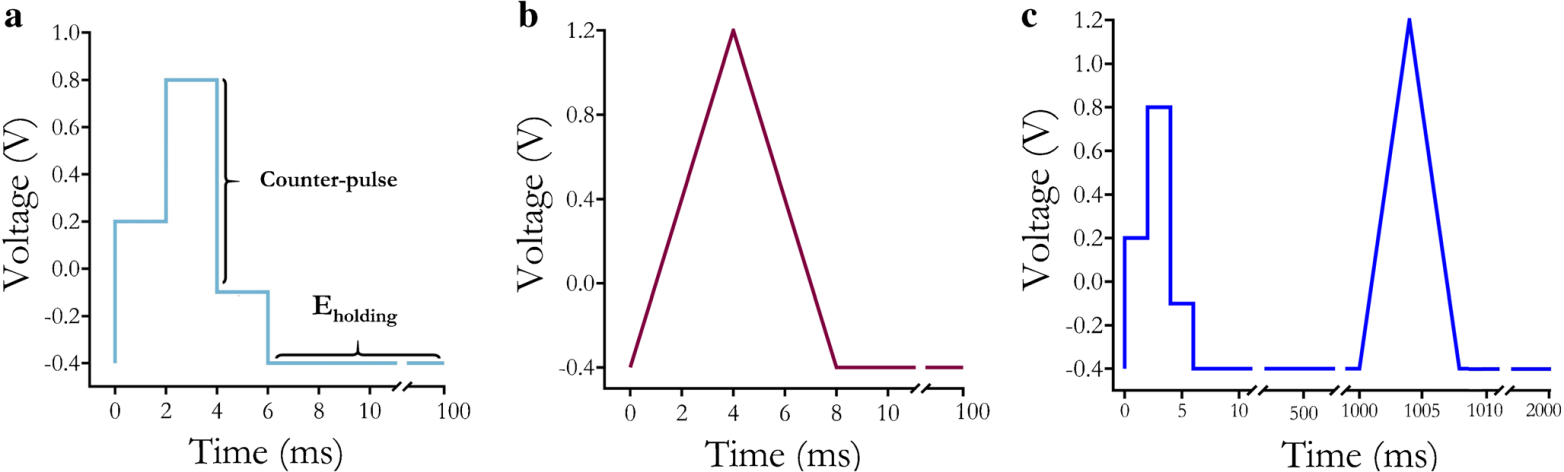
Voltammetry waveforms used in this study. **a**Four-step rapid pulse voltammetry (RPV) waveform. **b**Fast-scan cyclic voltammetry (FSCV) triangle waveform. **c** Combined RPV-FSCV waveform

Three different waveforms were used herein. (1) A four-step rapid pulse waveform consisting of −0.4 V to +0.2 V to +0.8 V to −0.1 V to −0.4 V at 2 ms per step applied at 10 Hz for in vitro RPV to investigate differentiating serotonin from dopamine (Fig. 1a). (2) A triangle waveform [41] for FSCV from −0.4 to +1.2 V to −0.4 V at a scan rate of 400 mV/s delivered at 10 Hz for in vitro comparisons with the RPV waveform (Fig. 1b). (3) A combination of the four-step rapid pulse and triangle waveforms described above. Each waveform was delivered in an alternating manner at 5 Hz in vivo and during post-calibration(Fig. 1c).

#### Machine learning

Data were extracted from raw MATLAB files into Excel and imported into Python using Pandas 0.25.1 and Jupyter 6.0.1 notebooks. All models were built using the Python 3.7.4 programming language in Jupyter notebooks using NumPy 1.16.5, SciPy 1.3.1, and scikit-learn 0.22.1 [81]. Data visualization was via matplotlib 3.1.1. Per each model built, data were normalized unless otherwise noted using either the *ℓ*_1_, *ℓ*_2_ or maximum norm, as chosen by grid search [81].

### Statistics

Statistical analyses for in vivo data (two-tailed *t*-tests; Table S1) were carried out using Prism, v.9.1.0 (GraphPad Inc., La Jolla, CA). Basal data over six timepoints just prior to the first optical stimulation were averaged for *N* = 3 microdialysis mice and *N* = 1 RPV mouse. The areas under the curve for microdialysis stimulation peaks were calculated using four dialysate samples after the onset of stimulation. Due to faster sampling, the areas under the curve for RPV stimulation peaks were calculated using fifty-two points post-stimulation onset. Data are expressed as means ± standard errors of the mean (SEMs). Throughout, *P* < 0.05 was considered statistically significant.

## Results and discussion

**Fig. 2:**
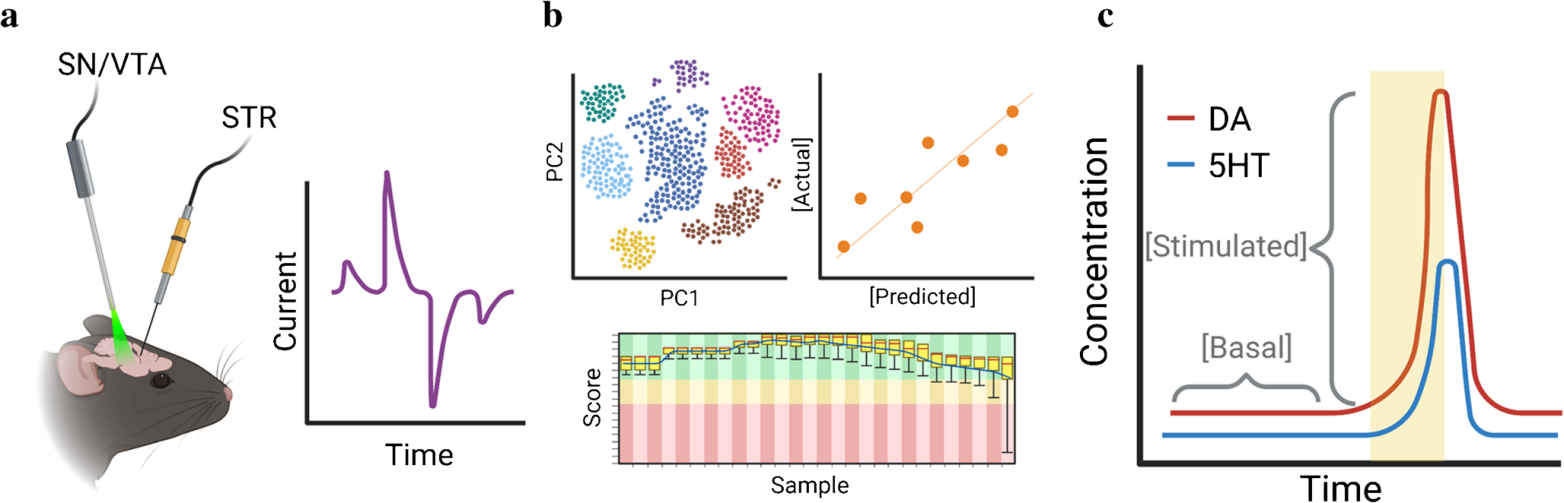
General scheme for rapid pulse voltammetry-partial least squares regression (RPV-PLSR). **a** Dopamine neurons in the substantia nigra and ventral tegmental area (SN/VTA) of DAT^IRE*Scre*^ mice were transfected with the excitatory opsin Chrimson. Basal and optically stimulated dopamine and serotonin levels were recorded from the striatum (STR) using the alternating RPV-fast-scan cyclic voltammetry waveform (Fig. 1c). **b** Electrodes used for in vivo measurements were then post-calibrated to provide data to build a PLSR model for analyte identification and quantification. **c** The in vivo data were analyzed using the model

We designed and evaluated an initial rapid pulse waveform in vitro for dopamine and serotonin co-detection(Fig. 1a) and to compare with a triangle waveform [41] (Fig. 1b). For in vivo experimentation, we alternated the rapid pulse and triangle waveforms (RPV-FSCV; Fig. 1c). Experimental paradigms utilizing these waveforms are shown in Fig. 2. The RPV-FSCV waveform was used to facilitate within-subjects’ comparisons (Fig. 2a). For experiments in mice, electrodes were post-calibrated in vitro to produce training set data (Fig. 2b). Training set data for each waveform were used to build machine learning regression models to classify and to quantify dopamine and serotonin (Fig. 2c). Multiple waveform-model combinations were compared in the context of cross-validation accuracy and predicted in vivo responses.

### Rapid pulse waveform design

We designed an initial rapid pulse waveform (Fig. 1a) based on potentials characteristic of commonly used dopamine or serotonin FSCV waveforms (Fig. 1b). The rapid pulse waveform employed a starting potential of −0.4 V, similar to a commonly used dopamine FSCV waveform [82] (Fig. 1b), but with steps to +0.2 V and −0.1 V, similar to the voltages scanned during the N-FSCV waveform used for preventing serotonin adsorption on electrode surfaces and to promote reduction of serotonin, respectively [83]. A step to +0.8 V was included to ensure the oxidation of serotonin and dopamine, while preventing capacitive currents from reaching the maximum current limits of our hardware, which occurs with large potential steps. Employing intermediate pulses (e.g., +0.2 V and −0.1 V) has been shown to increase analyte discrimination and precision for VETs [77]. Both faradaic and non-faradaic currents at intermediate steps contribute analyte-specific information more so than a single, large amplitude pulse step directly to the redox potential of interest (i.e., from −0.4 directly to +0.8 V), which would be dominated by capacitive current. In the future, intermediate steps can be added to reach +1.3 V, the upper potential commonly used for dopamine detection and recently optimized for serotonin detection [48]. This high upper potential was not used for this proof-of-concept experiment for simplification and to keep the pulse duration short. Employing a counter pulse completes the redox cycle and generates additional information on analyte identity, as demonstrated in electronic tongue pulse design [76].

### In vitro model construction

Data preprocessing is critical to the training and use of machine learning models, such as PLSR. Here, we use the terms “feature” or “variable” interchangeably to refer to the current response at a given time point in a voltammogram. We refer to a voltammogram as a “sample” determined using a particular combination of analyte concentrations (in vitro) or at a particular time relative to a stimulation event (in vivo).

Preprocessing typically involves mean-centering (setting means across all samples at each feature equal to zero) and either standardization (scaling the data to have unit variance at each feature across all samples) or normalization (scaling the input features to unit length). Mean-centering is done to simplify the computation process and should not affect model output [64]. Standardization is commonly used to remove magnitude-related effects, while normalization is used to preserve them. All are commonly accepted practices in the machine learning field, as well as for PLSR in chemometrics [64].

Previous implementations of FSCV with PCR [39] or PLSR [42] did not employ mean-centering or data standardization. By forgoing these procedures, the magnitudes of the original current responses were preserved. This caused the PCR or PLSR models to weigh regions of larger current amplitude (i.e., redox peaks) more heavily compared to low amplitude regions (i.e., noise). For techniques like FSCV, which rely mainly on variations in peak current responses for classification and quantification of analytes, non-standardized data make sense. The model should focus mostly on the variances at the highest peak magnitudes to correlate current magnitudes with concentration. However, pulse techniques, such as the VET and RPV, are explicitly designed *not* to rely solely on peak currents for quantification. Instead, the entire voltammogram is treated as a holistic source of predictive data. Thus, data are standardized, as the model should not treat larger current responses with greater importance.

**Fig. 3:**
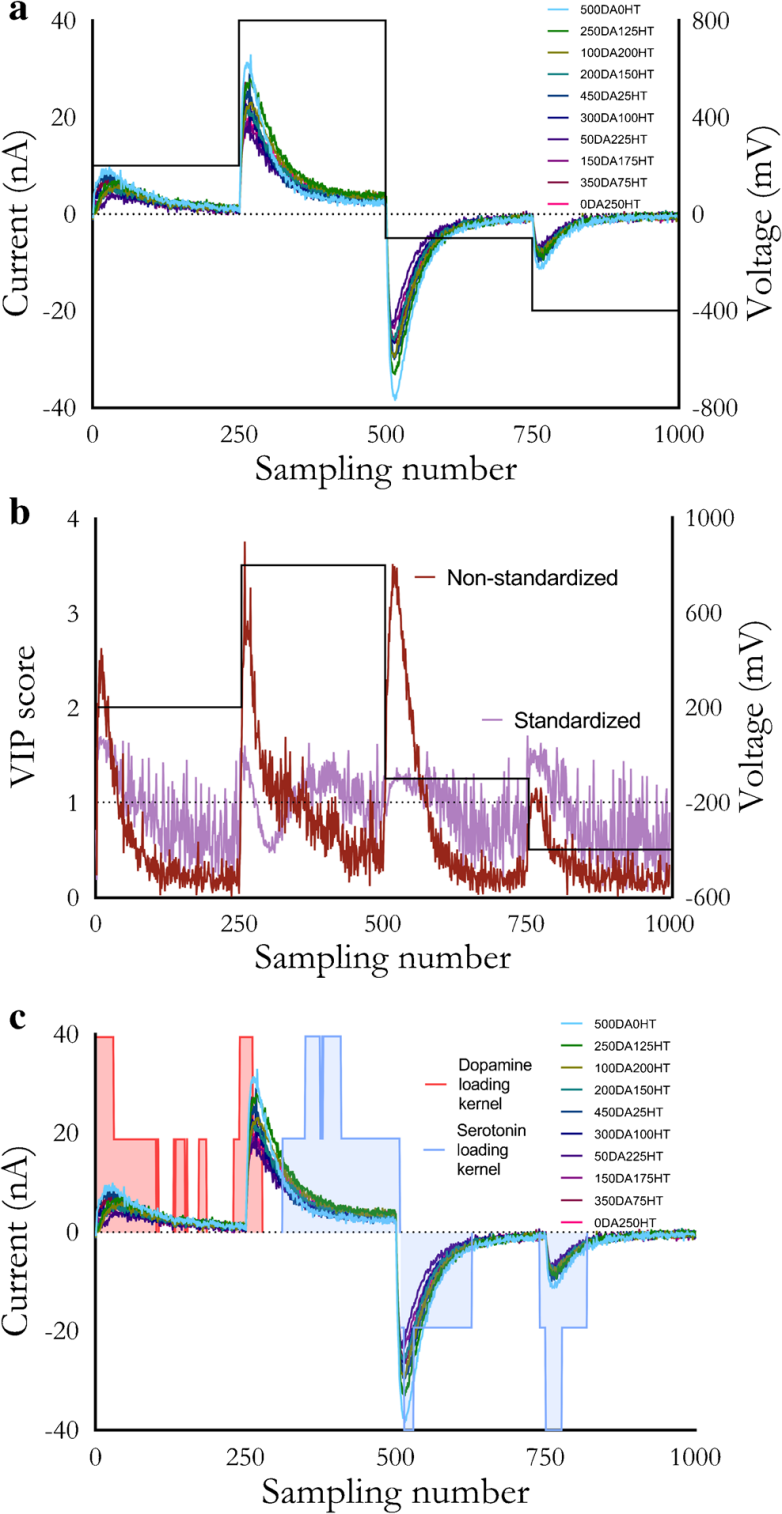
**a** Rapid pulse voltammograms of varying dopamine (DA) and serotonin (HT) combinations (nM). The pulse waveform is overlaid. **b** Variable importance in the projection (VIP) scores for non-standardized vs. standardized data obtained from **a**. **c** Loadings analysis overlaid with **a**

To investigate the effects of standardization on RPV data, we used a variable selection technique. The in vitro raw RPV voltammograms are shown in Fig. 3a. The samples obtained (1000 data points or “features”) were then represented in 1000 dimensions or principal components (PCs), each of which described some amount of variance in the data. The PCs were formed via a linear combination of the original variables and weighted projection coefficients, known as loadings [84]. Loading vectors of the greatest magnitude and similar direction in the factor space represented greater correlation.

Variable selection is the process of determining the features to present to the model as input. The relevance of different features can be examined through various methods based on the algorithm used. For PLSR, a variable importance in the projection (VIP) score can be mathematically calculated for each feature [85]. Generally, VIP scores >1 indicate variables that are important for the model to learn from the training data; features with scores <1 are considered less important. Thus, VIP scores can be used to evaluate waveform responses and serve three purposes. First, the VIP scores allow us to evaluate if RPV-PLSR is truly using current responses (features) not just from faradaic currents, but also from noise or capacitive currents. Second, the VIP scores allow us to evaluate how preprocessing affects feature importance (e.g., standardized vs. non-standardized data). And third, areas of the pulse response that are consistently more important for the model can be considered for more frequent sampling in future pulse designs, whereas areas of the current response that consistently have low VIP scores can be excluded by either reducing their sampling or removing that part of the pulse train. The VIP scores can be used as another metric to systematically optimize waveforms for a given analyte panel.

Preliminary analyses demonstrated that RPV-PLSR with standardized data are not dominated by magnitude-related effects and use areas of current response that historically have been discarded (Fig. 3b). For standardized data, the number of features with VIP scores >1 was 518 out of a possible 1000 features. For non-standardized data, the VIP scores clearly mimicked the magnitude of the current response. Moreover, the number of VIP scores >1 was only 231/1000. Standardizing the data allowed for a more than doubling of “important” features and these features spanned areas of the voltammogram dominated by non-faradaic current.

The use of non-faradaic current by the model is further supported by an analysis of the PLSR loadings (Fig. 3c). The magnitude of the projection of the **X** loading vectors onto the **Y** loading vectors was calculated as a mathematical representation of the strength of the correlation that each data point had with different combinations of dopamine and serotonin. To visualize regions of the voltammograms most informative for the model, a moving average kernel was applied to map each variable to low, medium, or high correlation (no shading, 50% shading, or 100% shading, respectively). Areas of the voltammograms with the highest shaded heights were most useful for that analyte (regardless of sign; positive or negative values are arbitrary). For example, the current response of the second pulse step (points 251–500) had high red-shaded areas during capacitive charging illustrating non-faradaic contributions to modeling dopamine. Meanwhile, the majority of the decay of the second pulse step, which would include faradaic and non-faradaic contributions, was heavily used for modeling serotonin.

Similar to the VIP scores (Fig. 3b), the loadings analysis (Fig. 3c)  demonstrates that RPV pulses can be optimized using PLSR analyses (e.g., the last two pulses could be shortened to improve temporal resolution as the tail-ends of each decay are not shaded). These findings support the theory behind intelligent (and iterative) pulse design in RPV and the key idea that background-subtracted methods, like FSCV, are likely to be inferior in terms of generating information needed to specify analytes and their concentrations, particularly in complex mixtures, because key information in capacitive current decay is removed. Instead, the VET-based approach used for RPV is a ‘soft’ technique, agnostically collecting information across the entire pulse train [86]. The capacitive current increases transiently and then decays exponentially due to the presence of charged and polar compounds. Concurrently, faradaic current approaches a limiting value based on the diffusion and adsorption rates of electroactive species. Using multivariate analysis, and specifically, dimensionality reduction, the model is trained on trends across the pulse train, not the response of individual currents, such as in univariate calibration [87].

Our findings are further supported by similar results for elastic net electrochemistry [88], in which the authors used non-background-subtracted voltammograms obtained using a FSCV triangular waveform to train an elastic net model, a regularized linear regression technique with some similarities to supervised dimensionality reduction techniques like PLSR [89]. The large magnitude (i.e., important) regularization coefficients, similar to the large magnitude loadings and VIP scores discussed above, were found to span areas of the voltammogram outside of the expected peak faradaic responses of dopamine and serotonin [66, 69].

A key difference between RPV and FSCV is that RPV is a pulse method having current decay across each pulse step. Since faradaic and capacitive currents evolve at different rates, each point in the decay provides unique information that is potentially useful for distinguishing analytes. That is, a stepped pulse approach is more information-rich when coupled with a regression model compared to a sweep method, even if background subtraction is bypassed in the latter. Furthermore, because RPV uses a bespoke pulse design, which can increase sensitivity when combined with electrode surface modifications [90], temporal resolution can be maximized by changing the pulse parameters. The waveform parameters in Fig. 1a are simply a starting point.

### In vivo model construction and deployment

Based on our preliminary in vitro RPV findings and the availability of suitable animal subjects from an ongoing study [11], we conducted a pilot in vivo study with RPV-PLSR. This small study was designed to compare the feasibility of an RPV waveform (that was admittedly unoptimized) with a commonly used FSCV waveform, early in our development of RPV and before continuing with the validation and creation of larger and more complex in vitro RPV training sets. We have found that advancing to in vivo experiments sooner in methods development helps to guide our in vitro efforts (sometimes in unexpected and fruitful ways) [91, 92].

We designed a combined rapid pulse-triangle waveform (RPV-FSCV) for use in conjunction with an optogenetic stimulation paradigm. The red-shifted opsin Chrimson was virally transfected into midbrain dopamine neurons in DAT^IRES*cre*^ mice. Four weeks later, carbon fiber microelectrodes coated with PEDOT:Nafion [79] were used to measure dopamine and serotonin in the striatum (STR). Optical stimulation (532 nm, 30 or 40 Hz, 20 s) was delivered to dopamine cell bodies in the substantia nigra/ventral tegmental area (SN/VTA) while the combined waveform (Fig. 1c) was applied to the carbon fiber microelectrode with each alternating waveform at 5 Hz. After several stimulations, the selective serotonin reuptake inhibitor (SSRI) escitalopram was administered, and the stimulation continued 1-h post-administration. Similar paradigms have been used to examine dopamine in STR [93]. Electrodes were then removed and used to obtain post-calibration training data for PLSR analysis (Table 1).

**Table 1:** Training set concentrations for in vivo post-calibration

Mouse	Injection	Dopamine (μM)	Serotonin (μM)
1	1	0.0	0.0
		5.0	0.0
	2	0.0	0.0
		0.0	4.0
	3	0.0	0.0
		2.0	2.0
2	1	0.0	0.0
		4.0	0.0
	2	0.0	0.0
		0.0	3.0
	3	0.0	0.0
		1.5	1.5
3	1	0.0	0.0
		5.0	0.0
	2	0.0	0.0
		0.0	5.0
	3	0.0	0.0
		2.0	2.0

Training set samples (one normalized, non-background-subtracted voltammogram per standard) were used to train and to cross-validate the PLSR model. While our hypothesis that standardization allows the model to place emphasis on response areas unrelated to magnitude was supported by our in vitro data (Fig. 3b), initial analyses of in vivo data using standardization resulted in negative predicted basal concentrations for dopamine, serotonin, or both. Nonetheless, dopamine and serotonin showed the expected qualitative and quantitative (nanomolar) responses to stimulation.

By removing magnitude-related effects via standardization, identification of analytes was possible, but quantitation became less reliable. We attributed this to the limited size and concentration range of the training set; standardization emphasizes variability. For accurate quantitation, standardization requires large data sets to train the model adequately on small-magnitude variations. Conversely, the VIP scores for the normalized data mimicked the non-standardized data in Fig. 3b, meaning that lower magnitude responses were still considered by the model due to inclusion of the background, but not as heavily as in standardization. Thus, normalization was used as the preprocessing method for the in vivo data to retain current amplitudes associated with a small training set size and for comparison to previous studies [63, 65].

After training the PLSR model, the number of components was optimized. The variance explained by the model is a function of the number of components included. For PLSR, the first component always explains the maximal covariance in the data, with successive decreases in covariance explained by additional components (i.e., the first component explains more covariance than the second, which explains more than the third, and so on). The total number of components equals the number of samples, at which point the data set is fully reconstructed (the cumulative variance explained reaches 100%). The model is then deployed with an a priori number of components such that only the most relevant features that lead to accurate analyte identification and quantification are used to make predictions, while the less relevant features (unrelated noise) are not utilized. Notably, “noise” as defined by background subtraction may differ from “noise” as defined by a PLSR model, meaning the background must be included to allow the PLSR model to discern the number of components. The number of components can be estimated based on training set conditions and domain knowledge (i.e., if the degrees of freedom of the system under study are known), or determined empirically, commonly by hyperparameter tuning during cross-validation.

To determine the variance in the *Y* variable (concentration) explained by the model, R^2^Y scores were calculated (Table 2). To estimate the generalizability of the model, Q^2^Y scores were calculated (i.e., cross-validated R^2^Y scores that serve as a proxy for predictive accuracy) using leave-one-outcross-validation because of the small training set size [94]. Given the known two-component calibration and variability of cross-validation errors for small training sets [95–97], we opted to deploy the two-component PLSR model in vivo at the expense of a lower in vitro cross-validation score (Q^2^Y = 0.1 for two components vs. Q^2^Y = 0.6 for three components). Although ostensibly detrimental to the model, selecting a model with higher cross-validation error can prevent overfitting, especially in the case of noisy training data [98]. The two-component model was used to predict in vivo concentrations of dopamine and serotonin simultaneously across time in a single subject.

**Table 2:** Training (R^2^Y) and cross-validation (Q^2^Y) accuracy metrics for each background-subtracted (no (N)/yes (Y))/waveform/model combination

**Model**	**Waveform**	**Background subtraction**		**R**^**2**^**Y**			**Q**^**2**^**Y**		
			*Components*	*2*	*3*	*5*	*2*	*3*	*5*
PLSR	Pulse	N		0.408	0.823	0.857	0.072	0.574	0.662
		Y		0.754	0.821	0.874	0.550	0.548	0.555
	Triangle	N		0.720	0.760	0.880	0.420	0.439	0.582
		Y		0.653	0.770	0.844	0.386	0.478	0.034
PCR	Pulse	N		0.356	0.421	0.876	0.033	−0.053	0.651
		Y		0.545	0.563	0.571	0.369	0.396	0.364
	Triangle	N		0.415	0.667	0.784	0.112	0.405	0.430
		Y		0.413	0.490	0.566	0.170	0.273	0.265

**Fig. 4:**
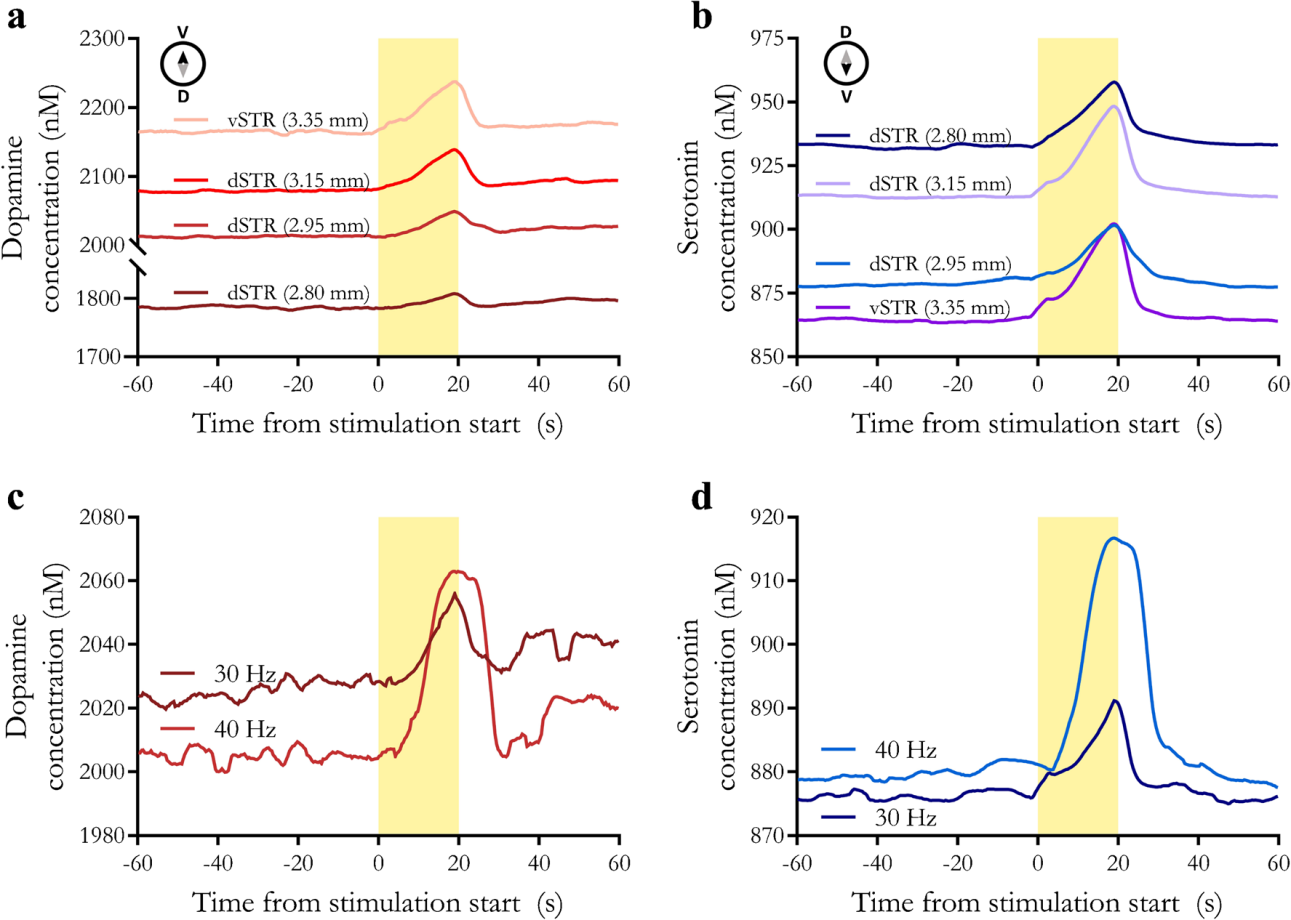
In vivo dopamine and serotonin monitoring using rapid pulse voltammetry with partial least squares regression (RPV-PLSR) analysis. **a**, **b** Time courses of dopamine or serotonin at various dorsoventral striatal positions measured with RPV-PLSR (*n* = 3 at 2.80 mm, *n* = 5 at 2.95 mm, *n* = 7 at 3.15 mm, and *n* = 3 at 3.35 mm for a total of 18 recordings in a single representative mouse). **c**, **d** Time courses of dopamine or serotonin measured in dorsal striatum (dSTR) in response to representative sequential 40 Hz and 30 Hz optical stimulations of midbrain dopamine neurons (*n* = 1)

As input to the RPV-PLSR model, for each stimulation, 300 scans (120 s total) were extracted that included 150 scans prior to stimulation (60 s) and 150 scans after the onset of stimulation (60 s). As output, the model predicted dopamine and serotonin concentrations for each scan based on the post-calibration training set. A moving average filter was applied to smooth and to align concentration vs. time plots. Basal concentrations were calculated as pre-stimulation baseline averages of the first 100 scans. Stimulated concentrations were defined as the areas under the curve for the stimulation peaks. Representative concentration-time plots are shown in Fig. 4.

During the experiment, the carbon fiber microelectrode was lowered from the dorsal striatum to the ventral striatum (dSTR and vSTR, respectively). Multiple stimulations were delivered at each position relative to the surface of the brain The average predicted basal concentration increased for dopamine and decreased for serotonin moving from dSTR to vSTR (Fig. 4a, b, respectively). These trends are in general agreement with previously reported dorsoventral dopamine and serotonin gradients in striatum [99], which is known to be neurochemically diverse [100]. To investigate the effects of stimulation strength, we applied a 40 Hz stimulation in the dorsal striatum and after ~5 min, applied a 30 Hz stimulation at the same electrode position. Higher frequency stimulation produced greater stimulated dopamine [101, 102] and serotonin release (Fig. 4c, d).

The predicted basal concentrations are most likely overestimates of actual concentrations given that we biased our in vivo training set towards higher dopamine and serotonin concentrations in this proof-of-concept study. Given this limitation, the relative differences of the simultaneous dopamine and serotonin levels under varying stimulation paradigms and model-waveform combinations are more important than absolute concentrations. Optical stimulation of dopamine neurons expressing the excitatory opsin Chrimson produced dopamine release detected by RPV-PLSR(Fig. 4a, c). The RPV-PLSR model, which was trained to differentiate dopamine and serotonin, also predicted serotonin release (Fig. 4b, d). Our recent microdialysis findings support the idea that optical stimulation of midbrain dopamine neurons produces serotonin release [11]. Linked dopamine and serotonin in the striatum have been reported elsewhere [60].

To increase our confidence in RPV-PLSR predictions, we compared the effects of serotonin transporter inhibition on basal and stimulated serotonin and dopamine using RPV-PLSR vs. microdialysis. The latter is a “gold standard” neurochemical monitoring method that relies on chromatographic separations for analyte identification and quantification [92, 103]. Similar to RPV, DAT^IRES*cre*^ mice were transfected with Chrimson for optical excitation of midbrain dopamine neurons during microdialysis [11]. Dialysis samples were collected at 5-min intervals and analyzed immediately online by HPLC with electrochemical detection. The optical stimulation was 5 min to match the dialysate sampling time. For RPV, we optically stimulated dopamine neurons for 20 s and sampled at 5 Hz.

**Fig. 5:**
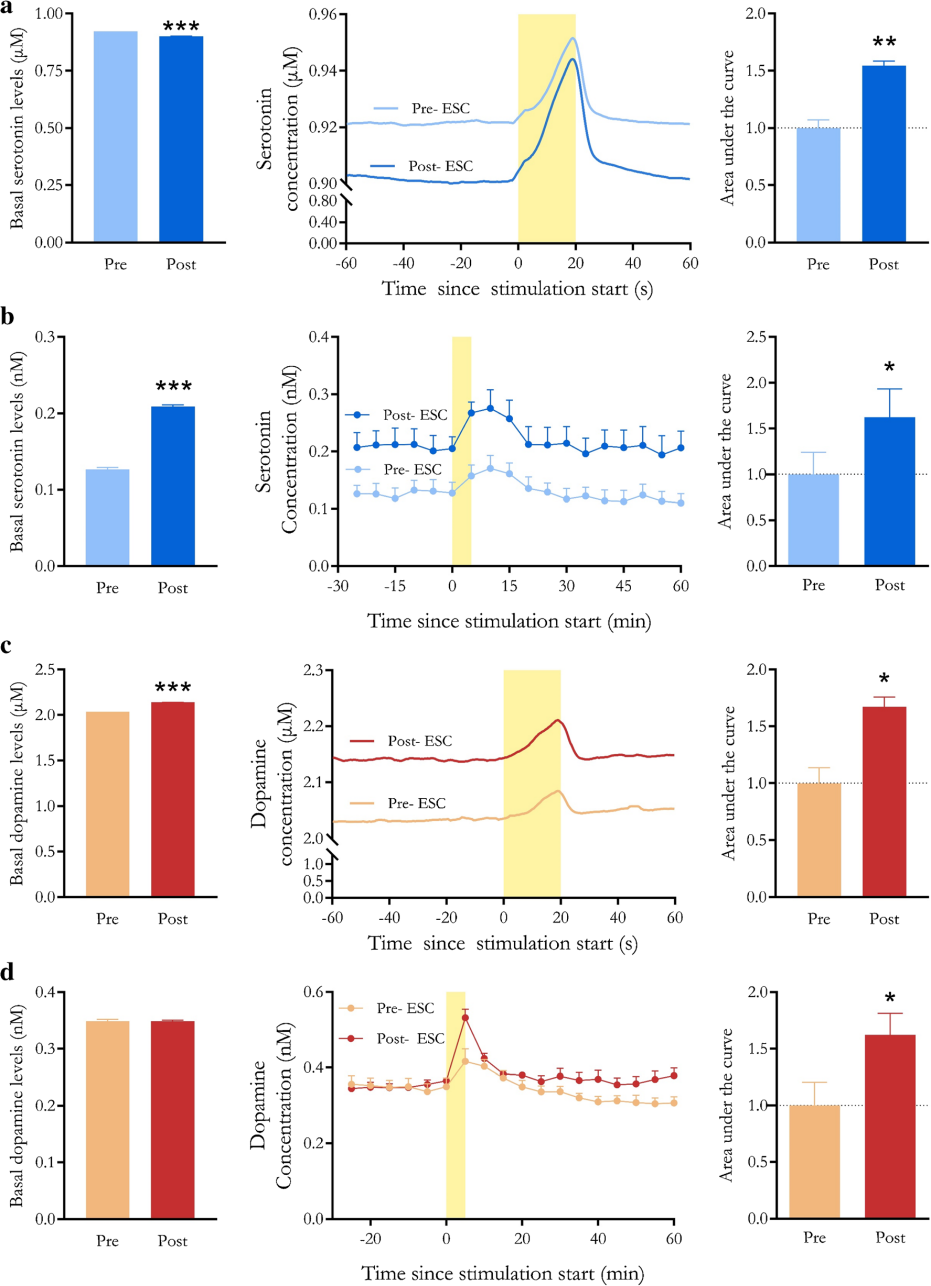
Responses to the selective serotonin reuptake inhibitor escitalopram by rapid pulse voltammetry with partial least squares regression analysis (RPV-PLSR) vs. microdialysis. Time courses are shown in the center panels for serotonin determined by **a**RPV-PLSR or **b** microdialysis and dopamine by **c**RPV-PLSR or **d** microdialysis. Escitalopram (20 mg/kg) was administered subcutaneously at *t* = −60 min for RPV-PLSR or perfused continuously into the dorsal striatum (10 μM) for microdialysis beginning at *t* = −90 min. Optical stimulation of Chrimson-transfected dopamine neurons occurred during the time periods marked by yellow bars. Basal serotonin or dopamine concentrations before and after/during escitalopram administration are shown in the left bar graphs. Stimulation-induced increases in serotonin or dopamine before vs. after/during escitalopram are shown in the right bar graphs and are calculated as areas under the curve. *P < 0.05, **P < 0.01, and ***P < 0.001 (see Table S1 and Methods for statistical details)

Following administration of the selective serotonin reuptake inhibitor (SSRI) escitalopram, we observed potentiation of optically evoked serotonin (i.e., greater area under the curve) determined by RPV-PLSR and microdialysis (Fig. 5a, b). Administration of an SSRI increases stimulated serotonin overflow due to reduced reuptake of serotonin by high-affinity serotonin transporters [58, 104, 105]. Serotonin reuptake inhibition also led to a 60% increase in basal serotonin levels [91] observed via microdialysis (Fig. 5b). By contrast, RPV-PLSR predicted a small relative decrease in basal extracellular serotonin (2%) (Fig. 5a).

One factor contributing to the RPV-PLSR prediction of lower basal serotonin following escitalopram involves the high concentration and limited number of standards used in the PLSR training set, which may result in insensitivity to modest changes. The RPV training set employed low micromolar concentration standards, whereas the predicted reduction in serotonin basal levels after escitalopram was only ~20 nM. Another factor potentially contributing to the discrepant effects of escitalopram on basal serotonin levels is the difference in the routes of drug administration. Mice in the microdialysis study received intrastriatal infusion of escitalopram, whereas mice in the RPV study were administered a subcutaneous drug injection. Systemic injection of an SSRI activates inhibitory 5HT1A autoreceptors on serotonin cell bodies [106, 107]. This negative feedback reduces serotonin neuron firing, which acutely results in reduced serotonin release in terminal regions like the striatum. Local infusion of escitalopram circumvents activation of somatodendritic 5HT1A receptors and produces an increase in terminal region serotonin levels [91].

Like serotonin, we observed escitalopram-induced potentiation of optically evoked dopamine by RPV-PLSR and microdialysis (Fig. 5c, d). Local perfusion of escitalopram did not affect basal dopamine levels determined by microdialysis (Fig. 5d), while subcutaneous injection of escitalopram was associated with a small (5%) increase in predicted basal dopamine levels by RPV-PLSR(Fig. 5c). As discussed, limitations of the training set used for RPV-PLSR, as well as the different routes of escitalopram administration, may underlie variations in the basal dopamine outcomes.

Despite the high selectivity of escitalopram for serotonin transporters and low affinity for dopamine transporters [108], the serotonin and dopamine systems are linked. Serotonin neurons innervate the SN and VTA, and both systems project to subcortical and cortical regions (e.g., striatum, frontal cortex, dorsomedial thalamus, cerebral cortex) [109, 110]. Serotonin receptors expressed on dopamine neurons in the striatum mediate dopamine release [111, 112]. Moreover, human imaging studies suggest that citalopram and/or escitalopram increase striatal dopamine levels [113] and dopamine transporter binding (as a compensatory response) [114, 115], presumably via increases in extracellular serotonin. Regardless of differences in absolute concentrations, microdialysis acts as external validation to confirm that optogenetic stimulation of dopamine neurons releases striatal serotonin and escitalopram potentiates optically stimulated dopamine. Overall, these findings indicate that RPV can be used to detect pharmacologically induced changes in the stimulated release of two neurotransmitters simultaneously in vivo.

### Waveform-model combination comparisons

To compare waveforms and analyses, R^2^Y and Q^2^Y scores were generated for different model/waveform/background-subtracted combinations using the in vivo post-calibration training set data (Table 1). In addition to two-component models, R^2^Y and Q^2^Y values were computed for three- and five-component models (Table 2) due to literature precedent [65]. Greater numbers of components were expected and found to produce erroneous results (negative concentrations, noisy oscillations), likely due to model overfitting. This supported our choice of the two-component model to analyze the in vivo results, rather than models with higher cross-validation scores [98]. However, due to the large increase in both R^2^Y and Q^2^Y moving from two to three components (an “elbow” point; see Fig. S3 ), three-component models were chosen to compare cross-validation scores across models. In all cases, training data were pre-processed with mean-centering and normalization.

We sought to answer three questions regarding RPV-PLSR in the context of the current training set data and to guide future studies. (1) How does RPV-PLSR compare, in terms of prediction, with previously developed FSCV-PCR (i.e., background-subtracted voltammograms obtained via a triangle waveform (Fig. 1b) and analyzed by PCR)? (2) Does including background current data in RPV-PLSR result in a benefit over background-subtracted RPV-PLSR, as suggested by Fig. 3c? (3) Does RPV-PLSR provide more information about analyte identification/quantification than FSCV-PCR or other possible combinations (e.g., why not use FSCV-PLSR?). We discuss various combinations below and find that each step of RPV-PLSR is needed to result in the optimal combination. For each combination, only the voltammograms for the relevant waveform were extracted to build the model (i.e., voltammograms from the triangle waveform were extracted when referring to FSCV; voltammograms from the pulse waveform were extracted when referred to RPV).

#### Comparing RPV-PLSR to FSCV-PCR

**Fig. 6:**
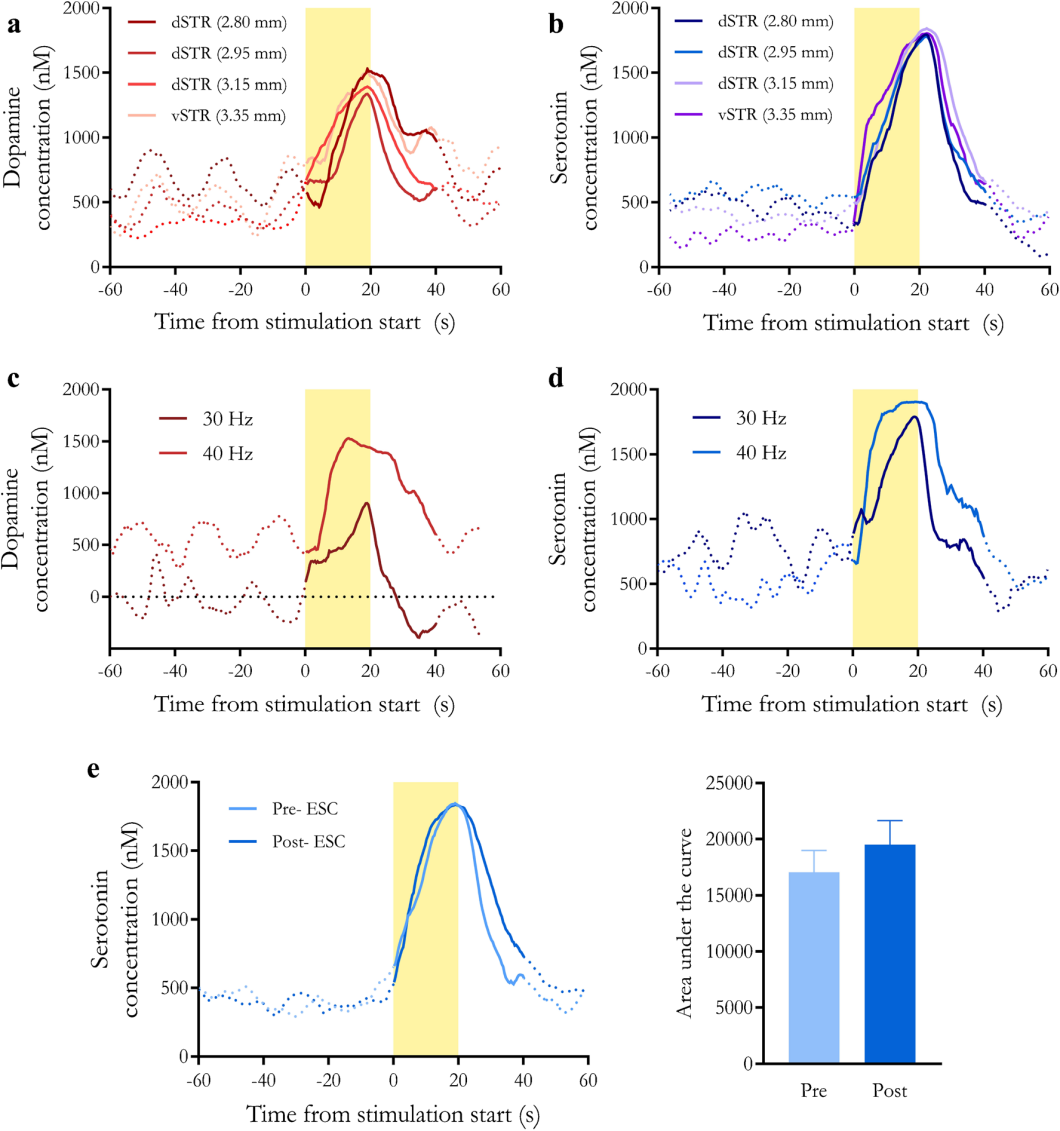
Predictions using a two-component fast-scan cyclic voltammetry-principal components regression (FSCV-PCR) model for dopamine and serotonin in vivo. **a**, **b** Time courses of dopamine and serotonin, respectively, at various dorsoventral striatal recording electrode positions determined by FSCV-PCR. **c**, **d** Time courses of dopamine and serotonin, respectively, in response to 40-Hz vs. 30-Hz stimulations predicted by FSCV-PCR. **e** Time course (left) and area under the curve (right) of serotonin for pre- and post-escitalopram administration using FSCV-PCR

Having demonstrated the non-background-subtractedRPV-PLSR waveform-model combination, the effects of striatal recording electrode position, optical stimulation frequency, and SSRI administration were examined using background-subtracted FSCV data and PCR analysis (Fig. 6). The FSCV-PCR model has been used for dopamine or serotonin monitoring [62]. Because background currents, which contain information about tonic neurotransmitters levels, are removed, the “basal” levels predicted by the FSCV-PCR model are not meaningful and, thus, were not considered.

Optically stimulated release of dopamine (Fig. 6a, c) and serotonin (Fig. 6b,d,e) were predicted by a two-component FSCV-PCR model. However, the stimulated concentrations were predicted to be much larger (~1 μM) than by RPV-PLSR and on the high end of literature reported values [34, 116, 117]. No increases in optically evoked release were detected in association with higher frequency stimulation for either dopamine or serotonin for FSCV-PCR analyses (Fig. 6c, d) or for serotonin following SSRI administration (Fig. 6e).

To ensure the model had enough components included to pick up on these differences, we tried increasing the number of components in the FSCV-PCR model from three to five. These additional components did not cause the serotonin traces to be distinguished by the stimulation paradigm (data not shown; i.e., the concentration traces looked the same for both 30 and 40 Hz stimulation frequency regardless of the number of components beyond two). This finding suggests that the model did have enough degrees of freedom, but was undertrained and consistently predicting a response that was not related to serotonin. Meanwhile, dopamine traces began to lose noticeable stimulation responses and showed increased noise as the number of components was increased from three to five, indicating that for this data set, two or three components appear to be better.

The results thus far support the notion that PLSR can deal more efficiently with noise and interferents when trained in vitro and used in vivo because PLSR models covariation of input and output, rather than just input, as in PCR. We did notice similarities in predicted responses for FSCV-PCR and FSCV-PLSR suggesting that overall, more training data and training across common interferents was needed. Furthermore, RPV-PCR produced similar traces in the same concentration range for dopamine compared to RPV-PLSR (1.8 to 2.3 μM). Serotonin traces showed more variation (larger SEMs) and slightly larger predicted concentrations (1.05 to 1.10 μM) but remained responsive to the stimulation paradigms. In both cases, stimulated responses were on the same order of magnitude as RPV-PLSR (10–100 nM). This is despite the low cross-validation score, again supporting the need to cautiously interpret these scores when small training sets are used. For these reasons, we could not state definitively the necessity for PLSR over PCR, other than to state that previous methods support the use of supervised learning over PCR for FSCV [66]. Because PLSR has been compared to PCR elsewhere [65, 84, 89], we do not compare results further here.

#### The need for including background current

We hypothesized that avoiding background subtraction would result in information gain for the RPV-PLSR model. We indeed observed greater cross-validation scores for non-background-subtracted compared to background-subtracted RPV-PLSR models (Table 2). However, this trend was not consistent across waveform-model combinations. The FSCV-PLSR and RPV-PCR analyses showed worse cross-validation accuracy without background subtraction (0.48 compared to 0.44 and 0.40 compared to −0.05, respectively), while the FSCV-PCR cross-validation score improved without background subtraction (0.27 compared to 0.41). Because no clear trend in cross-validation was present when background current was subtracted vs. not, we suspect that information gain may be waveform and model-dependent. Regardless, non-background-subtracted voltammograms obtained by our smart pulse waveform and analyzed by PLSR (i.e., RPV-PLSR) resulted in the highest three-component R^2^Y (0.82) and Q^2^Y (0.57) scores of all background/waveform/model combinations examined (Table 2). These variation and accuracy metrics suggest that RPV-PLSR may be better at modeling and predicting dopamine and serotonin concentrations, at least based on the limited training data.

#### Comparisons of additional waveform/model combinations

Other waveform/model/background subtraction combinations were explored (Table 2). Two-, three-, and five-component models were trained and used to analyze the in vivo post-calibration training set data. While RPV-PCR and FSCV-PLSR behaved somewhat similarly to RPV-PLSR and FSCV-PCR (Figs. S4, S5), in all other cases, except those discussed above, we did not find consistent, biologically relevant responses to stimulation paradigms (optical or pharmacological). Although it is possible these models would begin to produce meaningful results with more training data, we note that only the RPV-PLSR method worked reasonably well for this small data set. The RPV-PLSR method, compared to other waveform/model combinations, predicted the most reasonable relative differences when monitoring dopamine and serotonin across stimulation and pharmacologic paradigms. The absolute concentrations, however, should always be regarded as estimates, especially when using dimensionality reduction models [118]. Nonetheless, we attribute the success of RPV-PLSR to the wealth of information in the pulse and the parsimony of the PLSR model. When combined, our findings support the idea that RPV-PLSR can be used to extract maximally relevant information, even with small training set sizes.

### Study limitations and future directions

We note the following limitations of this proof-of-concept study. The first is training set size. While increased training set size should improve model generalizability [88, 119], training sets with similar sizes to ours (Table 1; *N* = 18) have been used in previous studies [66, 67]. The second limitation is the robustness of our training set. Notably, we did not train for responses to interferents (e.g., 5-hydroxyindoleacetic acid, 3,4-dihydroxyphenylacetic acid, ascorbic acid), changes in pH, or ionic salt concentrations (e.g., Na^+^, K^+^, Ca^2+^, Mg^2+^), any of which could conflate capacitive current responses in the PLSR model. This is a potential reason for the likely overestimated basal concentrations [120]. While our findings in vivo correspond with previously reported biological phenomena and relative trends, our basal concentrations are outside of what is expected for dopamine and serotonin based on previous voltammetry and microdialysis studies (~10–1000 nM and ~1–100 nM, respectively) [34, 56, 103, 121].

In the future, we plan to design more robust training sets that include interferents, pH changes, and ionic strength changes to investigate their influence on RPV-PLSR. However, most metabolites of dopamine and serotonin are not expected to change extracellularly (at least over short time frames) during stimulation because they are produced intracellularly [122–124]. Furthermore, because the RPV-PLSR model was trained using data across a four-step (i.e., intermediate) pulse voltammogram, it is less likely for the dimensionality reduction to confound interferents across multiple potential steps and time points. While varying pH was not considered in this training set, similar approaches have demonstrated pH insensitivity for dopamine and serotonin when using supervised learning, as opposed to unsupervised techniques (i.e., PCR) [66, 69].

Artifacts from ionic and pH changes during stimulated neurotransmitter release occur regardless of background subtraction [120, 125]. Some literature suggests that physiological changes in pH and divalent cationic salt concentrations may pose less of an interference problem for biogenic amines when using pulsed voltammetry [126], as opposed to FSCV, especially with Nafion-coated electrodes [127], potentially due to different surface binding mechanisms. The PEDOT:Nafion electrodes used here provide some selectivity against the anionic interferents mentioned above and reduce acute (6 h) biofouling [79], bolstering confidence in our predictions of cationic neurotransmitters.

Long-term (chronic) recordings can lead to variability in electrode responses due to biofouling. We will continue to calibrate multiple electrodes post-fouling (that is, after in vivo recording), which should account for some variability introduced over the course of brain implantation. We plan to increase the training size in future training sets, such that the model is trained on artifacts of fouling and other confounding factors mentioned above. We hypothesize that with increased training data, nonspecific signals can be parsed out by PLSR, or another supervised model. In theory, we could add short, highly anodic pulses (i.e., 1.3 V vs. Ag/AgCl) to try to renew electrode surfaces (electrochemical cleaning as employed in VETs [86] and FSCV [118]). Larger, historical training sets may also require ensemble weighting schemes to account for electrode variation [119].

At present, we do not directly compare RPV-PLSR to elastic net electrochemistry [88], another supervised learning technique. Theoretical comparisons of their underlying statistical approaches can be found elsewhere [89]. Instead, we note that dimensionality reduction techniques usually require less computation time than regularized techniques, suggesting that RPV-PLSR should scale well for larger training sets, which is a long-term goal of both techniques. However, both dimensionality reduction (PLSR) and regularization (elastic net) seek to prevent overfitting in some manner, whether by introducing sparsity in the latter case or by projecting data onto a lower-dimension feature space in the former. Thus, both methods improve robustness of predictions. The two methods can be combined as a form of variable selection due to their supervised nature (i.e., EN-PLS) [128, 129]. In fact, the RPV approach can theoretically be combined with any appropriate supervised regression technique that enables feature selection, representing a paradigm shift in the design and analysis of waveform-model combinations.

**Scheme 1:**
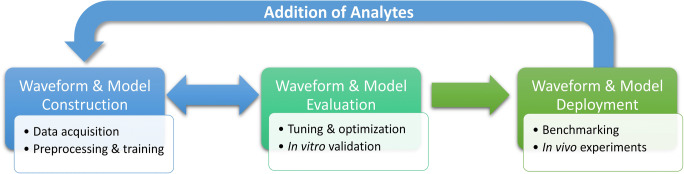
Rapid pulse voltammetry–machine learning optimization scheme

Based on the initial findings arising from the non-background-subtracted, supervised machine learning regression model (RPV-PLSR), we plan to optimize the pulse waveforms presented here, guided by feature selection as discussed earlier. Supervised learning techniques can enable iterative construction and optimization of fit-for-purpose waveforms to expand measurements to diverse sets of electroactive neurotransmitters (e.g., dopamine, serotonin, norepinephrine). We will also explore other pre-processing and feature selection techniques, as well as more advanced supervised regression models. Larger training sets across many electrodes with more diverse analyte/interferent panels will be needed [118]. Further validation and alternatives to in vitro training (i.e., relying on domain knowledge and stimulation paradigms, in addition to cross-validation metrics) should be explored to bolster confidence for in vivo predictions when using dimensionality reduction and regularization models that are trained and validated in vitro, but applied in vivo. Indeed, other areas of the physical sciences are currently working to address model generalizability through embeddings, representations, and domain knowledge [130]. Based on the current findings, future in vivo experiments can be designed more robustly to continue to investigate whether data processing models can distinguish and identify analytes in the complex brain matrix (e.g., validation using DAT inhibitors, dopamine and serotonin synthesis inhibitors, and in vivo standard addition). Overall, we foresee a new paradigm in which fit-for-purpose pulses are iteratively constructed with feature selection feedback (Scheme 1).

The coupling of voltammetry with more sophisticated pattern recognition and statistical tools is part of a global shift in scientific data analysis. Applications of machine learning in the physical sciences have skyrocketed over the last decade [131]. Other chemistry disciplines, such as materials, physical, and organic chemistry, were early adopters, but modern machine learning techniques have been underutilized in electrochemistry, and specifically voltammetry [132, 133]. While advanced techniques, such as deep learning, have been used for classification of voltammograms [134, 135], its counterpart (regression) is less often reported [88, 119]. The development of this novel voltammetric technique (RPV) coupled with fit-for-purpose machine learning (ML) pipelines (broadly defined as RPV-ML) represents a new paradigm for electroanalytical classification and quantitation of multiplexed neurochemical responses across timescales. This single, customizable technique allows for multiplexed neurotransmitter measurements in real time in behaving animals, representing a step towards decoding neurotransmission at the molecular scale.

## Conclusions

All three aspects of RPV appear essential for its success: intelligent pulse design, avoiding background subtraction, and supervised regression (i.e., PLSR). We have demonstrated that the RPV-PLSR combined paradigm can identify and quantify two neurotransmitters in vitro. When dopamine neurons were optically stimulated, the RPV-PLSR model detected serotonin release in vivo, which corroborates a novel finding by microdialysis using the same experimental paradigm (i.e., opsin, transfection and stimulation location, and recording location) [11]. Compared to FSCV-PCR and other waveform/model combinations, RPV-PLSR was better equipped to detect changes induced by different stimulation frequencies. When an SSRI was administered, RPV-PLSR detected increases in stimulated serotonin and dopamine levels, which were also corroborated by microdialysis. Overall, our experimental pipeline demonstrates proof-of-concept for a reliable new technique that can detect biologically relevant (i.e., nM) changes in basal and stimulated levels of multiple neurotransmitters simultaneously across biologically relevant timescales (i.e., stimulated and basal levels over ms to h).

## Supplementary Information

ESM 1 (PDF 3.46 MB)

## Data Availability

The datasets generated during and/or analyzed for the current study are available from the corresponding author (AMA) on reasonable request.

## Code availability

Code is available upon request.

## References

[1] Marcinkiewcz CA, Mazzone CM, D’Agostino G, Halladay LR, Hardaway JA, DiBerto JF, Navarro M, Burnham N, Cristiano C, Dorrier CE, Tipton GJ, Ramakrishnan C, Kozicz T, Deisseroth K, Thiele TE, McElligott ZA, Holmes A, Heisler LK, Kash TL (2016). Serotonin engages an anxiety and fear-promoting circuit in the extended amygdala. Nature..

[2] Hashemi P, Dankoski EC, Lama R, Wood KM, Takmakov P, Wightman RM (2012). Brain dopamine and serotonin differ in regulation and its consequences. Proc Natl Acad Sci U S A.

[3] Cheer JF, Heien MLAV, Garris PA, Carelli RM, Wightman RM (2005). Simultaneous dopamine and single-unit recordings reveal accumbens GABAergic responses: implications for intracranial self-stimulation. Proc Natl Acad Sci U S A.

[4] Ngernsutivorakul T, Steyer DJ, Valenta AC, Kennedy RT (2018). In vivo chemical monitoring at high spatiotemporal resolution using microfabricated sampling probes and droplet-based microfluidics coupled to mass spectrometry. Anal Chem.

[5] Tecuapetla F, Patel JC, Xenias H, English D, Tadros I, Shah F, Berlin J, Deisseroth K, Rice ME, Tepper JM, Koos T (2010). Glutamatergic signaling by mesolimbic dopamine neurons in the nucleus accumbens. J Neurosci.

[6] Amilhon B, Lepicard È, Renoir T, Mongeau R, Popa D, Poirel O, Miot S, Gras C, Gardier AM, Gallego J, Hamon M, Lanfumey L, Gasnier B, Giros B, El Mestikawy S (2010). VGLUT3 (vesicular glutamate transporter type 3) contribution to the regulation of serotonergic transmission and anxiety. J Neurosci.

[7] Mingote S, Chuhma N, Kalmbach A, Thomsen GM, Wang Y, Mihali A, Sferrazza C, Zucker-Scharff I, Siena A-C, Welch MG, Lizardi-Ortiz J, Sulzer D, Moore H, Gaisler-Salomon I, Rayport S (2017). Dopamine neuron dependent behaviors mediated by glutamate cotransmission. eLife..

[8] Root DH, Barker DJ, Estrin DJ, Miranda-Barrientos JA, Liu B, Zhang S, Wang H-L, Vautier F, Ramakrishnan C, Kim YS, Fenno L, Deisseroth K, Morales M (2020). Distinct signaling by ventral tegmental area glutamate, GABA, and combinatorial glutamate-GABA neurons in motivated behavior. Cell Rep.

[9] Wang H-L, Zhang S, Qi J, Wang H, Cachope R, Mejias-Aponte CA, et al. Dorsal raphe dual serotonin-glutamate neurons drive reward by establishing excitatory synapses on VTA mesoaccumbens dopamine neurons. Cell Rep. 2019;26(5):1128–42.e7. 10.1016/j.celrep.2019.01.014. 10.1016/j.celrep.2019.01.014 PMC6489450 30699344

[10] Lee K, Claar LD, Hachisuka A, Bakhurin KI, Nguyen J, Trott JM, Gill JL, Masmanidis SC (2020). Temporally restricted dopaminergic control of reward-conditioned movements. Nat Neurosci.

[11] Dagher M, Perrotta KA, Erwin SA, Hachisuka A, Ayer R, Bakhurin KI, Claar LD, Masmanidis S, Yang H, Andrews AM. Optogenetic stimulation of dopamine neurons induces serotonin co-transmission. Submitted for publication.

[12] Di Giovanni G, Esposito E, Di Matteo V (2010). Role of serotonin in central dopamine dysfunction. CNS Neurosci Ther.

[13] Aman TK, Shen R-Y, Haj-Dahmane S (2007). D2-like dopamine receptors depolarize dorsal raphe serotonin neurons through the activation of nonselective cationic conductance. J Pharmacol Exp Ther.

[14] Lee EHY, Geyer MA (1984). Dopamine autoreceptor mediation of the effects of apomorphine on serotonin neurons. Pharmacol Biochem Behav.

[15] Niederkofler V, Asher TE, Dymecki SM (2015). Functional interplay between dopaminergic and serotonergic neuronal systems during development and adulthood. ACS Chem Neurosci.

[16] Tan SKH, Hartung H, Schievink S, Sharp T, Temel Y (2013). High-frequency stimulation of the substantia nigra induces serotonin-dependent depression-like behavior in animal models. Biol Psychiatry.

[17] Altieri S, Singh Y, Sibille E, Andrews AM. Serotonergic pathways in depression. Neurobiology of Depression. 20115633: CRC Press; 2011. p. 143–70. 10.1201/b11232.

[18] Nestler EJ (2015). Role of the brain’s reward circuitry in depression: transcriptional mechanisms. Int Rev Neurobiol.

[19] Simpson EH, Kellendonk C, Ward RD, Richards V, Lipatova O, Fairhurst S, Kandel ER, Balsam PD (2011). Pharmacologic rescue of motivational deficit in an animal model of the negative symptoms of schizophrenia. Biol Psychiatry.

[20] Sumiyoshi T, Kunugi H, Nakagome K (2014). Serotonin and dopamine receptors in motivational and cognitive disturbances of schizophrenia. Front Neurosci.

[21] Rothman RB, Blough BE, Baumann MH (2007). Dual dopamine/serotonin releasers as potential medications for stimulante and alcohol addictions. AAPS J.

[22] Skowronek MH, Laucht M, Hohm E, Becker K, Schmidt MH (2006). Interaction between the dopamine D4 receptor and the serotonin transporter promoter polymorphisms in alcohol and tobacco use among 15-year-olds. Neurogenetics..

[23] Eskow Jaunarajs KL, George JA, Bishop C (2012). L-DOPA-induced dysregulation of extrastriatal dopamine and serotonin and affective symptoms in a bilateral rat model of Parkinson’s disease. Neuroscience..

[24] Stahl SM (2016). Parkinson’s disease psychosis as a serotonin-dopamine imbalance syndrome. CNS Spectr.

[25] Avery MC, Krichmar JL (2017). Neuromodulatory systems and their interactions: a review of models, theories, and experiments. Front Neural Circ.

[26] Zangen A, Nakash R, Overstreet D, Yadid G (2001). Association between depressive behavior and absence of serotonin-dopamine interaction in the nucleus accumbens. Psychopharmacology.

[27] Andrews AM (2013). The BRAIN initiative: toward a chemical connectome. ACS Chem Neurosci.

[28] Sarter M, Kim Y (2015). Interpreting chemical neurotransmission in vivo: techniques, time scales, and theories. ACS Chem Neurosci.

[29] Dreyer JK, Herrik KF, Berg RW, Hounsgaard JD (2010). Influence of phasic and tonic dopamine release on receptor activation. J Neurosci.

[30] Hajós M, Allers KA, Jennings K, Sharp T, Charette G, Sík A, Kocsis B (2007). Neurochemical identification of stereotypic burst-firing neurons in the rat dorsal raphe nucleus using juxtacellular labelling methods. Eur J Neurosci.

[31] Hajós M, Gartside SE, Villa AEP, Sharp T (1995). Evidence for a repetitive (burst) firing pattern in a sub-population of 5-hydroxytryptamine neurons in the dorsal and median raphe nuclei of the rat. Neuroscience..

[32] Hajós M, Sharp T (1996). Burst-firing activity of presumed 5-HT neurones of the rat dorsal raphe nucleus: electrophysiological analysis by antidromic stimulation. Brain Res.

[33] Sulzer D, Cragg SJ, Rice ME (2016). Striatal dopamine neurotransmission: regulation of release and uptake. Basal Ganglia.

[34] Abdalla A, Atcherley CW, Pathirathna P, Samaranayake S, Qiang B, Peña E, Morgan SL, Heien ML, Hashemi P (2017). In vivo ambient serotonin measurements at carbon-fiber microelectrodes. Anal Chem.

[35] Atcherley CW, Wood KM, Parent KL, Hashemi P, Heien ML (2015). The coaction of tonic and phasic dopamine dynamics. Chem Commun.

[36] Alivisatos AP, Andrews AM, Boyden ES, Chun M, Church GM, Deisseroth K, Donoghue JP, Fraser SE, Lippincott-Schwartz J, Looger LL, Masmanidis S, McEuen PL, Nurmikko AV, Park H, Peterka DS, Reid C, Roukes ML, Scherer A, Schnitzer M, Sejnowski TJ, Shepard KL, Tsao D, Turrigiano G, Weiss PS, Xu C, Yuste R, Zhuang X (2013). Nanotools for neuroscience and brain activity mapping. ACS Nano.

[37] Watson CJ, Venton BJ, Kennedy RT (2006). In vivo measurements of neurotransmitters by microdialysis sampling. Anal Chem.

[38] Bucher ES, Wightman RM (2015). Electrochemical analysis of neurotransmitters. Annu Rev Anal Chem.

[39] Su Y, Bian S, Sawan M (2020). Real-time in vivo detection techniques for neurotransmitters: a review. Analyst..

[40] Logman MJ, Budygin EA, Gainetdinov RR, Wightman RM (2000). Quantitation of in vivo measurements with carbon fiber microelectrodes. J Neurosci Methods.

[41] Singh YS, Sawarynski LE, Dabiri PD, Choi WR, Andrews AM (2011). Head-to-head comparisons of carbon fiber microelectrode coatings for sensitive and selective neurotransmitter detection by voltammetry. Anal Chem.

[42] Puthongkham P, Venton BJ (2020). Recent advances in fast-scan cyclic voltammetry. Analyst..

[43] Bunin MA, Prioleau C, Mailman RB, Wightman RM (1998). Release and uptake rates of 5-hydroxytryptamine in the dorsal raphe and substantia nigra reticulata of the rat brain. J Neurochem.

[44] Walters SH, Shu Z, Michael AC, Levitan ES (2020). Regional variation in striatal dopamine spillover and release plasticity. ACS Chem Neurosci.

[45] Nakatsuka N, Andrews AM (2017). Differentiating siblings: the case of dopamine and norepinephrine. ACS Chem Neurosci.

[46] Heien MLAV, Khan AS, Ariansen JL, Cheer JF, Phillips PEM, Wassum KM, Wightman RM (2005). Real-time measurement of dopamine fluctuations after cocaine in the brain of behaving rats. Proc Natl Acad Sci U S A.

[47] Venton BJ, Cao Q (2020). Fundamentals of fast-scan cyclic voltammetry for dopamine detection. Analyst..

[48] Dunham KE, Venton BJ (2020). Improving serotonin fast-scan cyclic voltammetry detection: new waveforms to reduce electrode fouling. Analyst..

[49] Atcherley CW, Laude ND, Parent KL, Heien ML (2013). Fast-scan controlled-adsorption voltammetry for the quantification of absolute concentrations and adsorption dynamics. Langmuir..

[50] West A, Best J, Abdalla A, Nijhout HF, Reed M, Hashemi P (2019). Voltammetric evidence for discrete serotonin circuits, linked to specific reuptake domains, in the mouse medial prefrontal cortex. Neurochem Int.

[51] Dengler AK, McCarty GS (2013). Microfabricated microelectrode sensor for measuring background and slowly changing dopamine concentrations. J Electroanal Chem.

[52] Kim SY, Oh YB, Shin HJ, Kim DH, Kim IY, Bennet K, Lee KH, Jang DP (2013). 5-hydroxytryptamine measurement using paired pulse voltammetry. Biomed Eng Lett.

[53] Meunier CJ, McCarty GS, Sombers LA (2019). Drift subtraction for fast-scan cyclic voltammetry using double-waveformpartial-least-squares regression. Anal Chem.

[54] Calhoun SE, Meunier CJ, Lee CA, McCarty GS, Sombers LA (2019). Characterization of a multiple-scan-rate voltammetric waveform for real-time detection of met-enkephalin. ACS Chem Neurosci.

[55] Meunier CJ, Mitchell EC, Roberts JG, Toups JV, McCarty GS, Sombers LA (2018). Electrochemical selectivity achieved using a double voltammetric waveform and partial least squares regression: differentiating endogenous hydrogen peroxide fluctuations from shifts in pH. Anal Chem.

[56] Oh Y, Heien ML, Park C, Kang YM, Kim J, Boschen SL, Shin H, Cho HU, Blaha CD, Bennet KE, Lee HK, Jung SJ, Kim IY, Lee KH, Jang DP (2018). Tracking tonic dopamine levels in vivo using multiple cyclic square wave voltammetry. Biosens Bioelectron.

[57] Park C, Oh Y, Shin H, Kim J, Kang Y, Sim J, Cho HU, Lee HK, Jung SJ, Blaha CD, Bennet KE, Heien ML, Lee KH, Kim IY, Jang DP (2018). Fast cyclic square-wave voltammetry to enhance neurotransmitter selectivity and sensitivity. Anal Chem.

[58] Shin H, Oh Y, Park C, Kang Y, Cho HU, Blaha CD, Bennet KE, Heien ML, Kim IY, Lee KH, Jang DP (2020). Sensitive and selective measurement of serotonin in vivo using fast cyclic square-wave voltammetry. Anal Chem.

[59] Swamy BEK, Venton BJ (2007). Carbon nanotube-modified microelectrodes for simultaneous detection of dopamine and serotoninin vivo. Analyst..

[60] Zhou F-M, Liang Y, Salas R, Zhang L, De Biasi M, Dani JA (2005). Corelease of dopamine and serotonin from striatal dopamine terminals. Neuron..

[61] Hermans A, Keithley RB, Kita JM, Sombers LA, Wightman RM (2008). Dopamine detection with fast-scan cyclic voltammetry used with analog background subtraction. Anal Chem.

[62] Heien MLAV, Johnson MA, Wightman RM (2004). Resolving neurotransmitters detected by fast-scan cyclic voltammetry. Anal Chem.

[63] Keithley RB, Mark Wightman R, Heien ML (2009). Multivariate concentration determination using principal component regression with residual analysis. Trends Anal Chem.

[64] Wold S, Sjöström M, Eriksson L (2001). PLS-regression: a basic tool of chemometrics. Chemom Intell Lab Syst.

[65] Kim J, Oh Y, Park C, Kang YM, Shin H, Kim IY, et al. Comparison study of partial least squares regression analysis and principal component analysis in fast-scan cyclic voltammetry. Int J Electrochem Sci. 2019;14(7):5924–37. 10.20964/2019.07.03.

[66] Kishida KT, Saez I, Lohrenz T, Witcher MR, Laxton AW, Tatter SB, et al. Subsecond dopamine fluctuations in human striatum encode superposed error signals about actual and counterfactual reward. Proc Natl Acad Sci U S A. 2016;113(1):200–5. 10.1073/pnas.1513619112. 10.1073/pnas.1513619112 PMC4711839 26598677

[67] Kishida KT, Sandberg SG, Lohrenz T, Comair YG, Sáez I, Phillips PEM, Montague PR (2011). Sub-second dopamine detection in human striatum. PLoS One.

[68] Bang D, Kishida KT, Lohrenz T, White JP, Laxton AW, Tatter SB, Fleming SM, Montague PR (2020). Sub-second dopamine and serotonin signaling in human striatum during perceptual decision-making. Neuron.

[69] Moran RJ, Kishida KT, Lohrenz T, Saez I, Laxton AW, Witcher MR, Tatter SB, Ellis TL, Phillips PEM, Dayan P, Montague PR (2018). The protective action encoding of serotonin transients in the human brain. Neuropsychopharmacology..

[70] Winquist F, Wide P, Lundström I (1997). An electronic tongue based on voltammetry. Anal Chim Acta.

[71] Campos I, Masot R, Alcañiz M, Gil L, Soto J, Vivancos JL, García-Breijo E, Labrador RH, Barat JM, Martínez-Mañez R (2010). Accurate concentration determination of anions nitrate, nitrite and chloride in minced meat using a voltammetric electronic tongue. Sensors Actuators B Chem.

[72] Labrador RH, Masot R, Alcañiz M, Baigts D, Soto J, Martínez-Mañez R, García-Breijo E, Gil L, Barat JM (2010). Prediction of NaCl, nitrate and nitrite contents in minced meat by using a voltammetric electronic tongue and an impedimetric sensor. Food Chem.

[73] Ivarsson P, Holmin S, Höjer N-E, Krantz-Rülcker C, Winquist F (2001). Discrimination of tea by means of a voltammetric electronic tongue and different applied waveforms. Sensors Actuators B Chem.

[74] Winquist F, Krantz-Rülcker C, Wide P, Lundström I (1998). Monitoring of freshness of milk by an electronic tongue on the basis of voltammetry. Meas Sci Technol.

[75] Ciosek P, Wróblewski W (2007). Sensor arrays for liquid sensing–electronic tongue systems. Analyst..

[76] Campos I, Alcañiz M, Masot R, Soto J, Martínez-Máñez R, Vivancos J-L, Gil L (2012). A method of pulse array design for voltammetric electronic tongues. Sensors Actuators B Chem.

[77] Fuentes E, Alcañiz M, Contat L, Baldeón EO, Barat JM, Grau R (2017). Influence of potential pulses amplitude sequence in a voltammetric electronic tongue (VET) applied to assess antioxidant capacity in aliso. Food Chem.

[78] Tian S-Y, Deng S-P, Chen Z-X (2007). Multifrequency large amplitude pulse voltammetry: a novel electrochemical method for electronic tongue. Sensors Actuators B Chem.

[79] Vreeland RF, Atcherley CW, Russell WS, Xie JY, Lu D, Laude ND, Porreca F, Heien ML (2015). Biocompatible PEDOT:Nafion composite electrode coatings for selective detection of neurotransmitters in vivo. Anal Chem.

[80] Sampson MM, Yang H, Andrews AM. Advanced microdialysis approaches resolve differences in serotonin homeostasis and signaling. Compendium of in vivo monitoring in real-time molecular neuroscience: WORLD SCIENTIFIC; 2017. p. 119–14010.1142/9789813220546_0005.

[81] Pedregosa F, Varoquaux G, Gramfort A, Michel V, Thirion B, Grisel O, Blondel M, Prettenhofer P, Weiss R, Dubourg V (2011). Scikit-learn: machine learning in Python. J Mach Learn Res.

[82] Heien MLAV, Phillips PEM, Stuber GD, Seipel AT, Wightman RM (2003). Overoxidation of carbon-fiber microelectrodes enhances dopamine adsorption and increases sensitivity. Analyst..

[83] Jackson BP, Dietz SM, Wightman RM (1995). Fast-scan cyclic voltammetry of 5-hydroxytryptamine. Anal Chem.

[84] Kramer R (1998). Chemometric techniques for quantitative analysis.

[85] Chong I-G, Jun C-H (2005). Performance of some variable selection methods when multicollinearity is present. Chemom Intell Lab Syst.

[86] Ivarsson P, Johansson M, Höjer N-E, Krantz-Rülcker C, Winquist F, Lundström I (2005). Supervision of rinses in a washing machine by a voltammetric electronic tongue. Sensors Actuators B Chem.

[87] Winquist F (2008). Voltammetric electronic tongues – basic principles and applications. Microchim Acta.

[88] Montague PR, Kishida KT (2018). Computational underpinnings of neuromodulation in humans. Cold Spring Harb Symp Quant Biol.

[89] Hastie T, Tibshirani R, Friedman JH (2001). The elements of statistical learning: data mining, inference, and prediction.

[90] Kawagoe KT, Zimmerman JB, Wightman RM (1993). Principles of voltammetry and microelectrode surface states. J Neurosci Methods.

[91] Yang H, Sampson MM, Senturk D, Andrews AM (2015). Sex- and SERT-mediated differences in stimulated serotonin revealed by fast microdialysis. ACS Chem Neurosci.

[92] Yang H, Thompson AB, McIntosh BJ, Altieri SC, Andrews AM (2013). Physiologically relevant changes in serotonin resolved by fast microdialysis. ACS Chem Neurosci.

[93] O’Neill B, Patel JC, Rice ME (2017). Characterization of optically and electrically evoked dopamine release in striatal slices from digenic knock-in mice with DAT-driven expression of channelrhodopsin. ACS Chem Neurosci.

[94] Martens HA, Dardenne P (1998). Validation and verification of regression in small data sets. Chemom Intell Lab Syst.

[95] Braga-Neto UM, Dougherty ER (2004). Is cross-validation valid for small-sample microarray classification?. Bioinformatics..

[96] Isaksson A, Wallman M, Göransson H, Gustafsson MG (2008). Cross-validation and bootstrapping are unreliable in small sample classification. Pattern Recogn Lett.

[97] Varoquaux G (2018). Cross-validation failure: small sample sizes lead to large error bars. NeuroImage..

[98] Ng AY. Preventing “overfitting” of cross-validation data. International Conference on Machine Learning (ICML); 1997: Citeseer.

[99] Zhang L, Doyon WM, Clark JJ, Phillips PE, Dani JA (2009). Controls of tonic and phasic dopamine transmission in the dorsal and ventral striatum. Mol Pharmacol.

[100] Brimblecombe KR, Cragg SJ (2017). The striosome and matrix compartments of the striatum: a path through the labyrinth from neurochemistry toward function. ACS Chem Neurosci.

[101] Hill DF, Parent KL, Atcherley CW, Cowen SL, Heien ML (2018). Differential release of dopamine in the nucleus accumbens evoked by low-versus high-frequency medial prefrontal cortex stimulation. Brain Stimul.

[102] Wightman RM, Amatorh C, Engstrom RC, Hale PD, Kristensen EW, Kuhr WG, May LJ (1988). Real-time characterization of dopamine overflow and uptake in the rat striatum. Neuroscience..

[103] Mathews TA, Fedele DE, Coppelli FM, Avila AM, Murphy DL, Andrews AM (2004). Gene dose-dependent alterations in extraneuronal serotonin but not dopamine in mice with reduced serotonin transporter expression. J Neurosci Methods.

[104] Daws LC, Toney GM, Davis DJ, Gerhardt GA, Frazer A (1997). In vivo chronoamperometric measurements of the clearance of exogenously applied serotonin in the rat dentate gyrus. J Neurosci Methods.

[105] Wood KM, Hashemi P (2013). Fast-scan cyclic voltammetry analysis of dynamic serotonin reponses to acute escitalopram. ACS Chem Neurosci.

[106] Dawson LA, Watson JM (2009). Vilazodone: a 5-HT1A receptor agonist/serotonin transporter inhibitor for the treatment of affective disorders. CNS Neurosci Ther.

[107] Gartside SE, Umbers V, Hajós M, Sharp T (1995). Interaction between a selective 5-HT1A receptor antagonist and an SSRI in vivo: effects on 5-HT cell firing and extracellular 5-HT. Br J Pharmacol.

[108] Owens MJ, Knight DL, Nemeroff CB (2001). Second-generation SSRIs: human monoamine transporter binding profile of escitalopram and R-fluoxetine. Biol Psychiatry.

[109] Conio B, Martino M, Magioncalda P, Escelsior A, Inglese M, Amore M, Northoff G (2020). Opposite effects of dopamine and serotonin on resting-state networks: review and implications for psychiatric disorders. Mol Psychiatry.

[110] Watabe-Uchida M, Zhu L, Ogawa Sachie K, Vamanrao A, Uchida N (2012). Whole-brain mapping of direct inputs to midbrain dopamine neurons. Neuron..

[111] Alex KD, Pehek EA (2007). Pharmacologic mechanisms of serotonergic regulation of dopamine neurotransmission. Pharmacol Ther.

[112] Navailles S, De Deurwaerdère P (2011). Presynaptic control of serotonin on striatal dopamine function. Psychopharmacology.

[113] Smith GS, Ma Y, Dhawan V, Chaly T, Eidelberg D (2009). Selective serotonin reuptake inhibitor (SSRI) modulation of striatal dopamine measured with [11C]-raclopride and positron emission tomography. Synapse..

[114] Warwick JM, Carey PD, Cassimjee N, Lochner C, Hemmings S, Moolman-Smook H, Beetge E, Dupont P, Stein DJ (2012). Dopamine transporter binding in social anxiety disorder: the effect of treatment with escitalopram. Metab Brain Dis.

[115] de Win MML, Habraken JBA, Reneman L, van den Brink W, den Heeten GJ, Booij J (2005). Validation of [123I]β-CIT SPECT to assess serotonin transporters in vivo in humans: a double-blind, placebo-controlled, crossover study with the selective serotonin reuptake inhibitor citalopram. Neuropsychopharmacology..

[116] Altieri SC, Yang H, O'Brien HJ, Redwine HM, Senturk D, Hensler JG, Andrews AM (2015). Perinatal vs genetic programming of serotonin states associated with anxiety. Neuropsychopharmacology..

[117] Hashemi P, Dankoski EC, Petrovic J, Keithley RB, Wightman RM (2009). Voltammetric detection of 5-hydroxytryptamine release in the rat brain. Anal Chem.

[118] Rodeberg NT, Sandberg SG, Johnson JA, Phillips PEM, Wightman RM (2017). Hitchhiker’s guide to voltammetry: acute and chronic electrodes for in vivo fast-scan cyclic voltammetry. ACS Chem Neurosci.

[119] Loewinger G, Patil P, Kishida KT, Parmigiani G. Multi-study learning for real-time neurochemical sensing in humans using the “study strap ensemble”. bioRxiv. 2021:856385. 10.1101/856385.

[120] Johnson JA, Hobbs CN, Wightman RM (2017). Removal of differential capacitive interferences in fast-scan cyclic voltammetry. Anal Chem.

[121] Gardier AM, David DJ, Jego G, Przybylski C, Jacquot C, Durier S, Gruwez B, Douvier E, Beauverie P, Poisson N, Hen R, Bourin M (2003). Effects of chronic paroxetine treatment on dialysate serotonin in 5-HT1B receptor knockout mice. J Neurochem.

[122] Meiser J, Weindl D, Hiller K (2013). Complexity of dopamine metabolism. Cell Commun Signal.

[123] Mohammad-Zadeh LF, Moses L, Gwaltney-Brant SM (2008). Serotonin: a review. J Vet Pharmacol Ther.

[124] Qi Z, Miller GW, Voit EO, Wellstead P, Cloutier M (2012). Mathematical models of dopamine metabolism in Parkinson’s disease. Systems biology of Parkinson's disease.

[125] Takmakov P, Zachek MK, Keithley RB, Bucher ES, McCarty GS, Wightman RM (2010). Characterization of local pH changes in brain using fast-scan cyclic voltammetry with carbon microelectrodes. Anal Chem.

[126] Yoshimi K, Weitemier A (2014). Temporal differentiation of pH-dependent capacitive current from dopamine. Anal Chem.

[127] Gerhardt GA, Hoffman AF (2001). Effects of recording media composition on the responses of Nafion-coated carbon fiber microelectrodes measured using high-speed chronoamperometry. J Neurosci Methods.

[128] Fu G-H, Xu Q-S, Li H-D, Cao D-S, Liang Y-Z (2011). Elastic net grouping variable selection combined with partial least squares regression (EN-PLSR) for the analysis of strongly multi-collinear spectroscopic data. Appl Spectrosc.

[129] Giglio C, Brown SD (2018). Using elastic net regression to perform spectrally relevant variable selection. J Chemom.

[130] Vasudevan RK, Ziatdinov M, Vlcek L, Kalinin SV (2021). Off-the-shelf deep learning is not enough, and requires parsimony, Bayesianity, and causality. npj Comput Mater.

[131] Carleo G, Cirac I, Cranmer K, Daudet L, Schuld M, Tishby N, Vogt-Maranto L, Zdeborová L (2019). Machine learning and the physical sciences. Rev Mod Phys.

[132] Gundry L, Guo S-X, Kennedy G, Keith J, Robinson M, Gavaghan D, Bond AM, Zhang J (2021). Recent advances and future perspectives for automated parameterisation, Bayesian inference and machine learning in voltammetry. Chem Commun.

[133] Bond AM (2020). A perceived paucity of quantitative studies in the modern era of voltammetry: prospects for parameterisation of complex reactions in Bayesian and machine learning frameworks. J Solid State Electrochem.

[134] Matsushita GHG, Sugi AH, Costa YMG, Gomez-A A, Da Cunha C, Oliveira LS (2019). Phasic dopamine release identification using convolutional neural network. Comput Biol Med.

[135] Ye J-J, Lin C-H, Huang X-J (2020). Analyzing the anodic stripping square wave voltammetry of heavy metal ions via machine learning: information beyond a single voltammetric peak. J Electroanal Chem.

