# Supplementary material for: Simultaneous serotonin and dopamine monitoring across timescales by rapid pulse voltammetry with partial least squares regression
Source: Anal Bioanal Chem. 2021 Oct 23;413(27):6747–67. doi: 10.1007/s00216-021-03665-1 (PMC8551120; doi:10.1007/s00216-021-03665-1)
Supplement: Supplementary file 1 — (PDF 3.46 MB) [file 216_2021_3665_MOESM1_ESM.pdf]

Supplemental information for:

Simultaneous serotonin and dopamine monitoring across timescales by rapid pulse voltammetry with partial least squares regression

Cameron S. Movassaghi,[1,2] Katie A. Perrotta,[1] Hongyan Yang,[3]

Rahul Iyer,[4] Xinyi Cheng,[1] Merel Dagher,[5]

Miguel Alcañiz Fillol,[6]* and Anne M. Andrews[1,2,3,5]*

[1]*Department of Chemistry & Biochemistry, University of California, Los Angeles, Los Angeles, CA 90095, USA*

[2]*California NanoSystems Institute, University of California, Los Angeles, Los Angeles, CA 90095, USA*

[3]*Department of Psychiatry and Biobehavioral Sciences, Semel Institute for Neuroscience and Human Behavior, and Hatos Center for Neuropharmacology, University of California, Los Angeles, Los Angeles, CA 90095, USA*

[4]*Department of Electrical Engineering, University of California, Los Angeles, Los Angeles, CA 90095, USA*

[5]*Molecular Toxicology Interdepartmental Program, University of California, Los Angeles, Los Angeles, CA 90095, United States*

[6]*Interuniversity Research Institute for Molecular Recognition and Technological Development, Universitat Politècnica de València - Universitat de València, Camino de Vera s/n, 46022 Valencia, Spain*

*Correspondence to mialcan@upvnet.upv.es and aandrews@mednet.ucla.edu

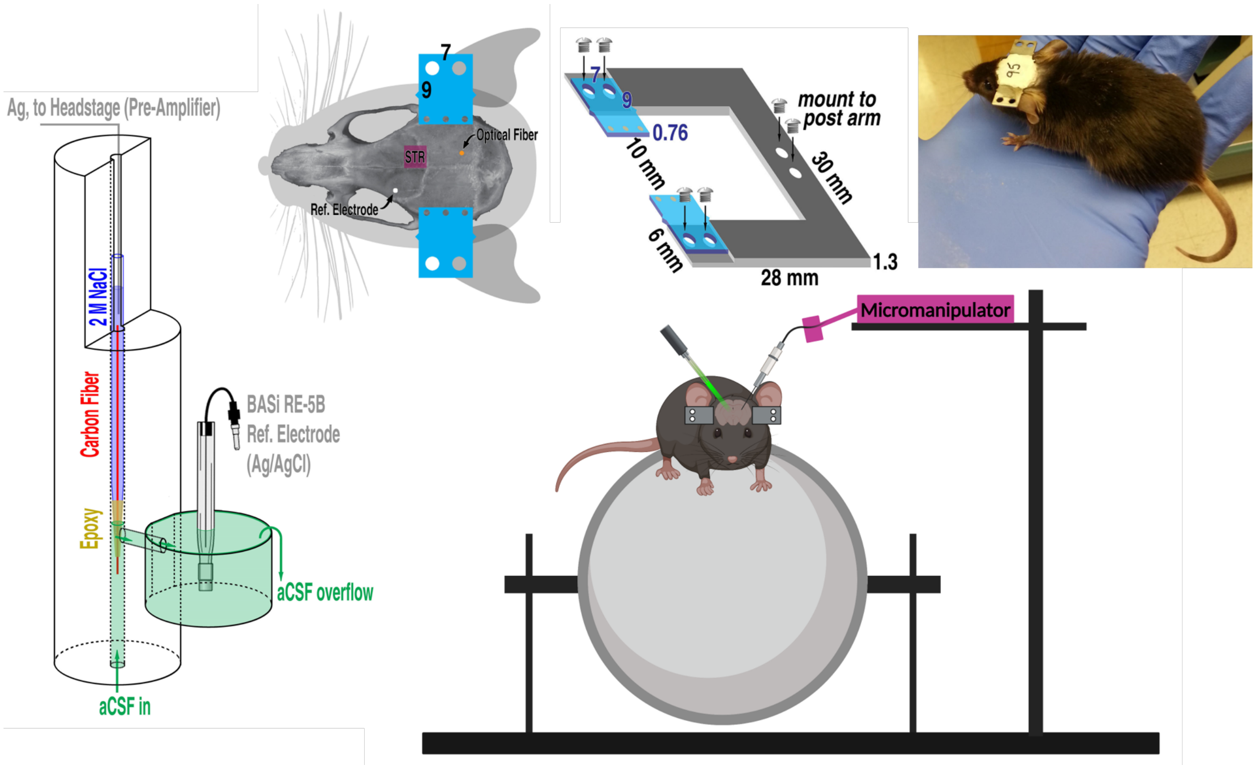

Figure S1. Experimental set-up for *in vitro* carbon-fiber microelectrode calibration (flow cell, left) and *in vivo* experiments (right). Photograph courtesy of Wesley Smith.

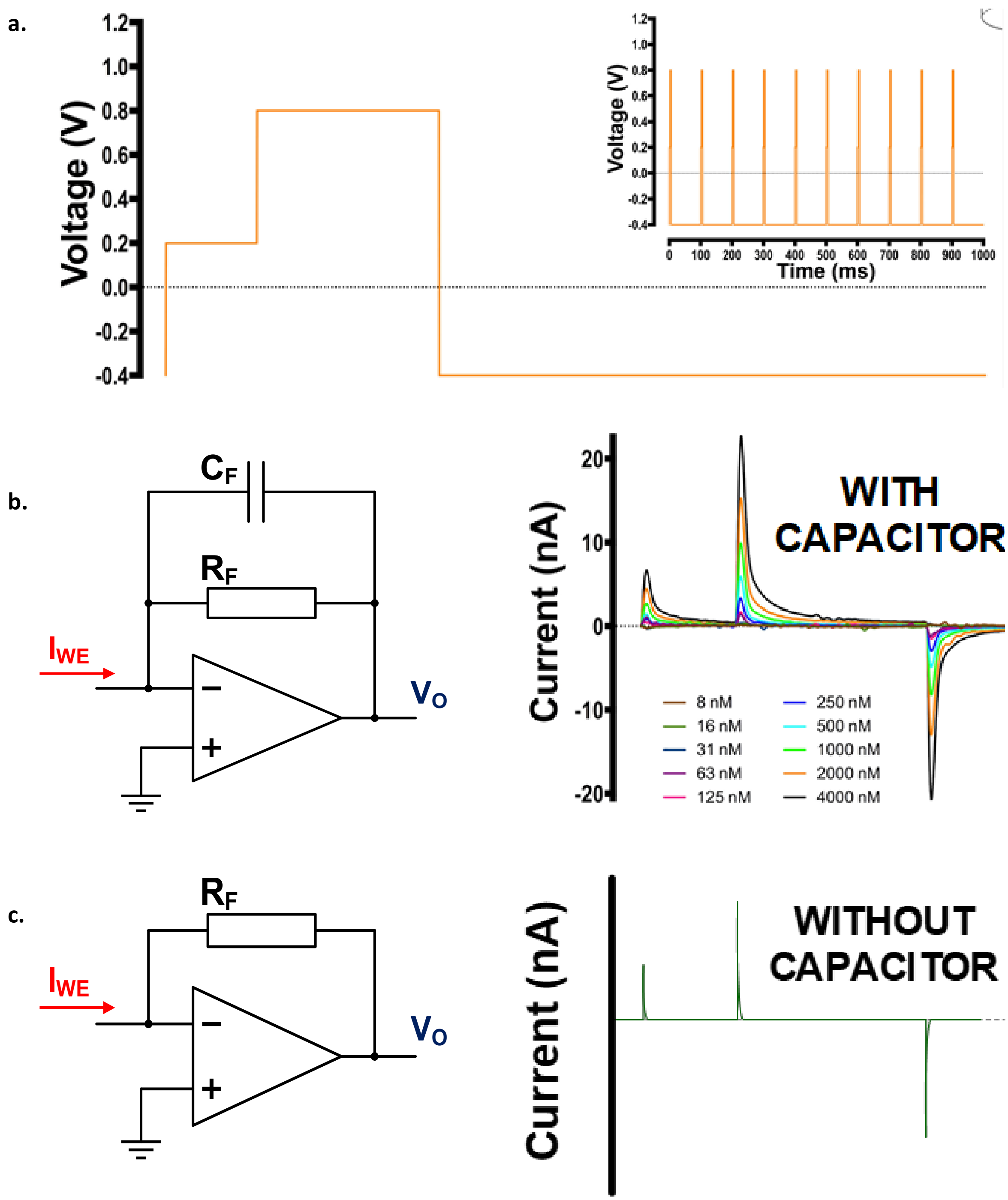

Figure S2. Effect of the transimpedance amplifier used in the custom headstage. **(a)** Representative pulse applied to the electrochemical system. **(b)** Circuit diagram for the transimpedance amplifier (amplifier with feedback resistor R_F in parallel with feedback capacitor C_F) to convert current from the working electrode (I_{WE}) into output voltage (V_O) (left). Representative voltammograms with the capacitor in parallel (right). **(c)** Circuit diagram for amplifier without feedback capacitor (left). Representative voltammogram (right).

The rapid pulse voltammetry (RPV) waveform is shown in **Figure S2a**. For this circuit (**Fig. S2b**), the relationship between the output voltage and the working electrode current is given by the differential equation:

$$\frac{V_O}{R_F} + C_F \cdot \frac{dV_O}{dt} = -I_{WE} \quad \textbf{(1)}$$

If the feedback capacitor is not present (**Fig. S2c**), the relationship is given by:

$$V_O = -R_F \cdot I_{WE} \quad \textbf{(2)}$$

Transimpedance amplifiers often include a feedback capacitor connected in parallel to the feedback resistor for signal stabilization and filtering purposes [S1,S2]. The drawback of their use is that the feedback capacitor disrupts the direct proportionality between measured current and output voltage (**Equation 1**). Instead, the output signal will deform (*i.e.,* the exponential current decay will be delayed) according to the differential present in Equation 1 (**Fig. S2b**). In most cases, the latter is not desired.

For RPV, we purposefully included a feedback capacitor (**Fig. S2b**) to allow a smoother, longer duration of the pulse response. Important electrochemical information is present within the nonfaradaic and faradaic currents immediately after a pulse, on the order of tens of microseconds. Without a feedback capacitor, these currents decay too quickly to be sampled with high enough time resolution by the data acquisition system (8 µs sampling rate; 125 kHz). With a feedback capacitor of appropriate capacitance, the output voltage response is spread out over a longer temporal duration. This additional decay time affords the PLSR model more data points to be sampled from key electrochemical events that would otherwise be missed or under-sampled. While we recognize these electronic components preclude the analysis of an electrochemical system by equivalent circuit analysis, due to the deformation of the original working electrode signal, we note our purpose here is to obtain relevant information for the PLSR model, not to gain mechanistic insights at the electrode surface. Furthermore, the capacitor helps to stabilize and to reduce the noise of the output signal.

For these reasons, we implemented the feedback capacitor as shown in **Figure S2b**. The value of the capacitor was determined empirically to produce the desired smoothing and decay time of the signal, depending on the pulse sequence applied. For our set up, this value was found to be approximately 100 pF.

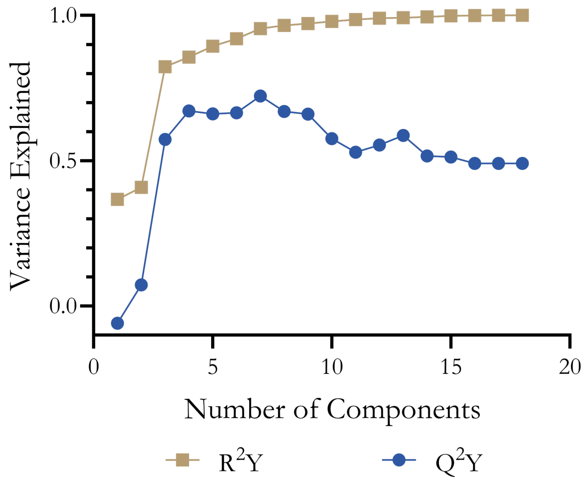

Figure S3. Cumulative training (R^2Y) and prediction (Q^2Y) score metrics for the RPV-PLSR model with respect to the number of components.

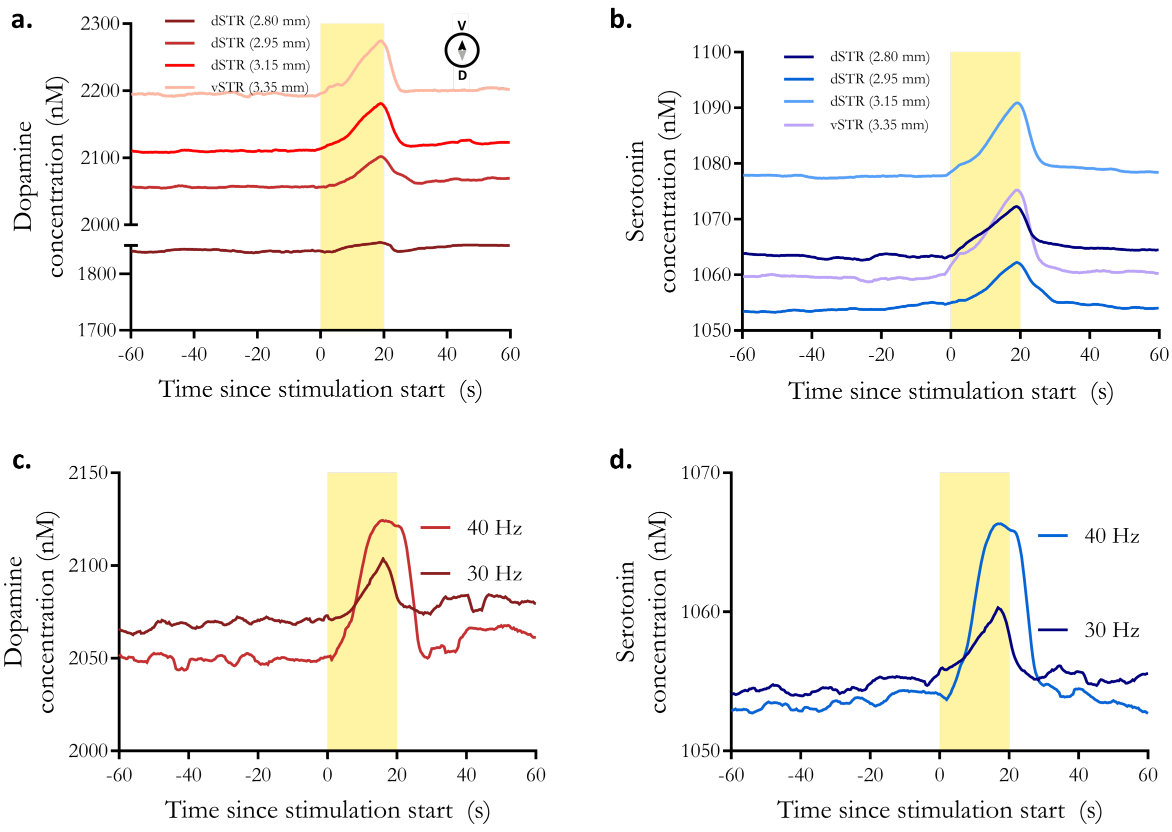

Figure S4. *In vivo* dopamine and serotonin monitoring using rapid-pulse voltammetry with principal components regression (RPV-PCR) analysis. (a,b) Time courses of dopamine or serotonin at various dorsoventral striatal positions measured with RPV-PCR. **(c,d)** Time courses of dopamine or serotonin measured in dorsal striatum (dSTR) in response to representative 40 Hz or 30 Hz sequential optical stimulations of midbrain dopamine neurons.

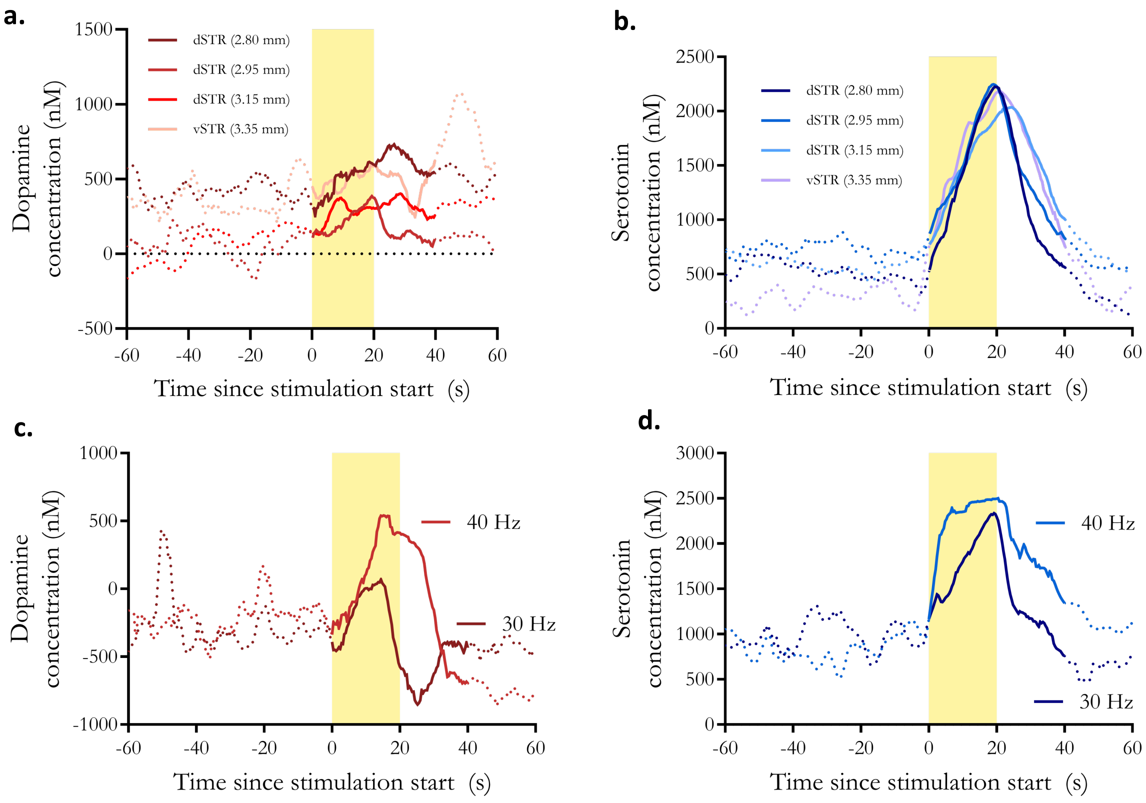

Figure S5. Predictions using a FSCV-PLSR model for dopamine and serotonin *in vivo.*
(a,b) Time courses of dopamine and serotonin, respectively, at various dorsoventral striatal recording electrode positions determined by FSCV-PLSR. **(c,d)** Time courses of dopamine and serotonin, respectively in response to 30 Hz *vs.* 40 Hz stimulations predicted by FSCV-PLSR.

Table S1. Statistical summary.

Figure	Comparison	Test	Statistics	Significant?
5A, left	Basal 5HT RPV: pre- SSRI *vs.* post- SSRI	Paired *t*-test	t(5)=238	*P*<0.001
5A, right	AUC 5HT RPV: pre- SSRI *vs.* post- SSRI	Unpaired *t*-test	t(5)=5.92	*P*<0.01
5B, left	Basal 5HT microdialysis: pre- SSRI *vs.* post- SSRI	Paired *t*-test	t(4)=21.7	*P*<0.001
5B, right	AUC 5HT microdialysis: pre- SSRI *vs.* post- SSRI	Paired *t*-test	t(8)=2.67	*P*<0.05
5C, left	Basal DA RPV: pre- SSRI *vs.* post- SSRI	Paired *t*-test	t(5)=544	*P*<0.001
5C, right	AUC DA RPV: pre- SSRI *vs.* post- SSRI	Unpaired *t*-test	t(5)=3.823	*P*<0.05
5D, left	Basal DA microdialysis: pre- SSRI *vs.* post- SSRI	Paired *t*-test	t(4)=0.030	ns
5D, right	AUC DA microdialysis: pre- SSRI *vs.* post- SSRI	Paired *t*-test	t(8)=2.843	*P*<0.05

The *t*-tests were two-tailed and paired or unpaired depending on whether matching numbers of pre- *vs.* post SSRI samples were available.

Supplemental Citations

S1. Orozco L (2013) Programmable-gain transimpedance amplifiers maximize dynamic range in spectroscopy systems. Analog Dialogue. https://www.analog.com/en/analog-dialogue/articles/programmable-gain-transimpedance-amplifiers.html. Accessed May 14 2021

S2. Bhat A (2012) Stabilize transimpedance amplifier circuit design (application note 5129). Maxim Integrated. https://www.maximintegrated.com/en/design/technical-documents/app-notes/5/5129.html. Accessed May 14 2021
